# Feasibility Study on the Use of NO_2_ and PM_2.5_ Sensors for Exposure Assessment and Indoor Source Apportionment at Fixed Locations

**DOI:** 10.3390/s24175767

**Published:** 2024-09-05

**Authors:** Miriam Chacón-Mateos, Erika Remy, Uta Liebers, Frank Heimann, Christian Witt, Ulrich Vogt

**Affiliations:** 1 Department of Flue Gas Cleaning and Air Quality Control, University of Stuttgart, 70569 Stuttgart, Germany; ulrich.vogt@ifk.uni-stuttgart.de; 2 Institute of Physics and Meteorology, University of Hohenheim, 70599 Stuttgart, Germany; 3 Institute of Physiology, Charité—Universitätsmedizin Berlin, Corporate Member of Freie Universität Berlin and Humboldt-Universität zu Berlin, 10117 Berlin, Germanychristian.witt@charite.de (C.W.); 4 Department of Pneumology, Evangelische Lungenklinik Berlin Buch, 13125 Berlin, Germany; 5 Ambulante Pneumologie mit Allergie Zentrum, 70178 Stuttgart, Germany

**Keywords:** air quality, low-cost sensors, indoor air, exposure assessment, source apportionment, I/O ratio

## Abstract

Recent advances in sensor technology for air pollution monitoring open new possibilities in the field of environmental epidemiology. The low spatial resolution of fixed outdoor measurement stations and modelling uncertainties currently limit the understanding of personal exposure. In this context, air quality sensor systems (AQSSs) offer significant potential to enhance personal exposure assessment. A pilot study was conducted to investigate the feasibility of the NO_2_ sensor model B43F and the particulate matter (PM) sensor model OPC-R1, both from Alphasense (UK), for use in epidemiological studies. Seven patients with chronic obstructive pulmonary disease (COPD) or asthma had built-for-purpose sensor systems placed inside and outside of their homes at fixed locations for one month. Participants documented their indoor activities, presence in the house, window status, and symptom severity and performed a peak expiratory flow test. The potential inhaled doses of PM_2.5_ and NO_2_ were calculated using different data sources such as outdoor data from air quality monitoring stations, indoor data from AQSSs, and generic inhalation rates (IR) or activity-specific IR. Moreover, the relation between indoor and outdoor air quality obtained with AQSSs, an indoor source apportionment study, and an evaluation of the suitability of the AQSS data for studying the relationship between air quality and health were investigated. The results highlight the value of the sensor data and the importance of monitoring indoor air quality and activity patterns to avoid exposure misclassification. The use of AQSSs at fixed locations shows promise for larger-scale and/or long-term epidemiological studies.

## 1. Introduction

Air pollution has long been known to affect health, and it contributes heavily to the Global Burden of Disease [1,2]. As stated in the United Nations Sustainable Development Goals, improving air quality is a pillar of improving global health and well-being (Goal 3), as well as creating safe and sustainable cities (Goal 11) [3]. However, as it stands now, approximately 6.7 million deaths can be attributed to air pollution each year [4]. The most recent WHO air quality guidelines describe air pollution as “the single biggest environmental threat to human health” [5].

The number of epidemiological studies showing the health effects of air pollution has been growing in recent years, but it is far from being complete [6]. With more research emerging, the effects of both long- and short-term exposure to pollutants are now better understood. It is also recognised that the level of exposure at which harmful long-term health problems can occur is significantly lower than was thought before [5,7]. 

The current standard to measure air quality relies on permanent outdoor monitoring stations. While these stations provide accurate and continuous measurements, both the equipment-purchase and maintenance costs are high. Due to their expense, their spatial distribution across the world is limited [8]. According to Fuller et al. [4], most urban areas in Europe and North America have at least one measurement station, which corresponds to one station for every 100,000 to 500,000 people, approximately. Due to the high spatial variability of pollutant concentrations, the existing distribution of monitoring stations is inadequate for accurately measuring air quality across different microenvironments [7,9].

Standard outdoor monitoring stations leave another gap in understanding air quality’s relation to health, in that only ambient outdoor air is measured. The majority of the population in developed countries spends most of their time in indoor environments [10]. Bulot et al. [11] report that indoor pollution exposure causes around half of all pollutant-related deaths and that the associated risks “cannot be accurately studied by outdoor monitoring stations”. Any individual’s exposure to pollution cannot be fully described without understanding both the indoor and outdoor air quality [12].

As with many problems linked to climate change, there is a clear disparity in pollution exposure across the globe [13,14]. Low- and middle-income countries (LMICs) bear the brunt of deaths and economic loss due to air pollution [4]. Even as conditions improve in some wealthier countries, air quality continues to deteriorate in many LMICs [15]. According to the WHO, this is in part due to economic development in LMICs being reliant on fossil fuel burning [5]. Not only is there global inequality in exposure but also in resources available to effectively measure pollution levels [16,17]. Sub-Saharan Africa is reported by Fuller et al. [4] to have roughly one permanent measurement station per 15.9 million people. This extreme imbalance further emphasizes the need for alternative measurement methods [18].

Air quality sensor systems (AQSSs) for monitoring air quality have shown potential as practical solutions to these needs [19,20,21,22]. Current research in this field is focused on two main approaches: the dynamic or direct approach, which utilizes portable sensors, and the static or indirect approach, which relies on stationary sensors. The difference in cost and the portability of smaller sensors allow studies that track individual exposure, in contrast to the bulkier reference-grade instruments. For example, a study using wearable sensors on bicycles demonstrated that the health benefits associated with cycling could be partially offset by exposure to traffic-related air pollution along the route [23]. Moreover, multipollutant AQSSs have been developed and tested for estimating individual-level pollutant doses with promising results [24,25,26]. However, these studies also highlighted the limitations and significant resources required for evaluating in-transit or commuting data.

The widespread use of AQSSs has also enhanced research on exposure assessment across different microenvironments. A study conducted on a university campus in Beijing, China, used fixed AQSSs to investigate the impact of outdoor-origin PM_2.5_ on potential inhaled doses indoors [27]. Amegah et al. [28] conducted a study at traffic hotspots in Accra (Ghana) using Purple Air PA-II monitors and self-reported health questionnaires and found consistent evidence that PM_2.5_ exposure among street traders increases the occurrence of respiratory and cardiovascular symptoms.

The increased spatio-temporal resolution provided by AQSS networks has the potential to improve our understanding of individual exposure pathways [29]. The future of environmental health studies lies in the integration of big data from sensors and models. Research on data fusion has already explored both the dynamic and static approaches. For instance, recent studies have demonstrated that combining stationary AQSSs with dispersion models can enhance assessments of pollutant exposure, including human mobility patterns [30,31,32]. Other researchers have focused on integrating model data with information from wearable sensors [33,34], enabling more accurate exposure assessments and better health risk management.

Epidemiological studies will benefit immensely from the enhanced spatial resolution enabled by AQSSs. In particular, resource-limited regions can find in AQSSs a solution to close the data gap left by the scarce or non-existent monitoring and help address air quality management [35]. As stated by Vilcassim and Thurston [7], AQSSs will be a key part of “more democratized, high resolution, and inter-connected air (and health) monitoring, generating ‘big data’ for complex, but more inclusive, research”.

In this study, a pilot project was carried out to evaluate the feasibility of using AQSSs at fixed locations for epidemiological investigations. This study aimed to evaluate the use of AQSSs over a longer timeframe, which so far has not been thoroughly studied [36,37]. To this end, custom-built stationary sensor systems for NO_2_ and PM_2.5_ were deployed for approximately 30 days both in- and outside the homes of seven individuals diagnosed with asthma or chronic obstructive pulmonary disease (COPD). Participants recorded their indoor daily activities as well as the window status and home presence on an hourly basis. Using this information alongside the AQSS data, we analysed the activity-specific indoor and outdoor NO_2_ and PM_2.5_ ratio (I/O ratio) and conducted indoor source apportionment. Additionally, indoor activities were classified into four intensity levels and the activity adjusted inhalation rates (IR) were calculated based on them. This information has been used to evaluate the exposure misclassification of the potential NO_2_ and PM_2.5_ dose. Comparisons of the calculated potential doses were made between using outdoor air quality monitoring stations or indoor AQSS data, and using generic IR versus activity adjusted IR. The results were compared with other studies, and a comprehensive discussion of the challenges encountered during the study was included as well. Finally, a qualitative symptomatology analysis was conducted to explore the suitability of the AQSS data for studying the relationship between air quality and health. The health data used in this analysis were self-reported by participants using a health questionnaire that we developed. 

## 2. Methods

### 2.1. Study Design and Population

This study took place in Stuttgart, the sixth largest city in Germany with an estimated population of 630,000. Stuttgart has a unique and complicated meteorological and urban climate since the city centre is set in a basin surrounded by hills, and only opens to the northwest, where it meets the Neckar River Valley [38]. The geographical situation, combined with the fact that the busy main roads traverse the basin, often leads to temperature inversions during the cold months, which in turn worsens the air quality in the city centre [39].

The measurement campaign lasted approximately six months, spanning from winter to spring. The first measurements were taken on 19 December 2019 and the last measurements on 28 May 2020. The indoor and outdoor AQSSs were deployed in the patient’s home for approximately one month.

The AQSSs were checked once in the middle of the measurement period to ensure that everything was working properly and that data were still being collected. The dates when AQSSs were deployed and checked in each patient’s home are detailed in Table S1 in the Supplementary Materials.

Personnel of the pulmonology practice assisted in recruiting patients to participate in this study. The criteria for a patient to qualify for participation in this study included, among others, being aged 18 years and over, being diagnosed with COPD or asthma, not requiring supplemental oxygen therapy, and living near a busy road in Stuttgart. Seven adult subjects of varying ages and diagnoses participated in this study. The demographics of the patients are shown in Table S2. Patients provided written consent for their participation, and all collected data were kept pseudo-anonymous. Individuals are referred to by a patient identification number (ID). This number was assigned by the doctor to maintain patient anonymity. In this manuscript, the participants are referred to by numbers from 1 to 7.

### 2.2. Materials

Two AQSSs were designed, tailored to their usage either indoors or outdoors (see Figure S1 in the Supplementary Materials). The NO_2_ and PM_2.5_ sensors were from the company Alphasense (Great Notley, UK), model B43F and OPC-R1, respectively. Additionally, a temperature and relative humidity (RH) sensor model HYT221 from the company IST (Ebnat-Kappel, Switzerland) was included in both sensor systems. The indoor AQSSs were positioned in the participants’ living room, chosen based on patients’ feedback indicating it as their primary location for daily activities. Patients 1, 2, 3, 6, and 7 each had a single outdoor AQSS placed by their house. For patient 4, two AQSSs were placed outside on opposite sides of the house. One AQSS was positioned on the street-facing side of the house, while the other faced towards the garden. From here on, to differentiate between the two outdoor AQSSs for patient 4 they are referred to as either “street” or “garden”. Outdoor data for patient 2 were lost due to a heavy storm. Patient 5 did not provide consent for an outdoor AQSS. The patients were unable to view the sensor data during the measurement period to avoid prompting changes in their everyday routines. 

The air quality monitoring station operated by the University of Stuttgart at Hauptstätter Street was used for co-location. This station represents a traffic hotspot and is located in the city centre. The approximate locations of the patients’ homes and the outdoor monitoring station are shown in Figure S1 in the Supplementary Materials.

### 2.3. Data Collection

A summary of the data collected (or excluded) for each patient is shown in Table S3. At the start of the experiment, each participant’s home was mapped using observations from an in-person visit, and together with the patient, an environmental survey about the house, the daily routines, the building, and the area was conducted. Examples of information from the environmental survey include the type of landscape around the home, the distance from the nearest busy road, the type of stove, and whether there are additional residents in the home. Selected results from these environmental surveys are shown in Table S4 for all participants. 

Patients were instructed to complete a logbook, recording hourly information across three distinct categories: patient location, activities at home, and room environment. The environmental category specifically included details such as window status (open, closed, or tilted), air conditioning use, and the presence of a functioning fireplace. However, none of the patients reported having either air conditioning or a fireplace in operation. Therefore, the analysis presented in this manuscript focuses solely on the window status within the room environment category. An example logbook is provided in Figure S2.

The category “activities at home”, also called activity log, included ten common indoor activities. As activities were tracked each hour, sometimes multiple activities were recorded during the same hour. There were also some hours for which no data were provided. In the data analysis, several default values were selected to account for instances where a patient did not report any activities. The default values were set to “home”, “unknown”, and “window closed” accordingly. The three grouping categories, their possible values, and the default value are given in Table 1. Patients who had a garden or balcony (2, 3, 4, 5, 6, and 7) were asked to log when they used it during the day. For patients who had additional residents in their homes (3, 5, 6, 7), the activities of the additional residents were not recorded. 

Participants were also asked to complete a daily health survey about the presence and severity of respiratory symptoms. Another key method to monitor asthma and COPD patient’s health is to check lung function. For that purpose, each patient was given a peak flow meter, shown how to use it properly, and asked to self-test their peak expiratory flow (PEF) each evening. PEF is the rate at which air exits the lungs in one quick and forceful exhale and is given in units of L min^−1^. 

### 2.4. Data Analysis

#### 2.4.1. Overview

**Figure 1:**
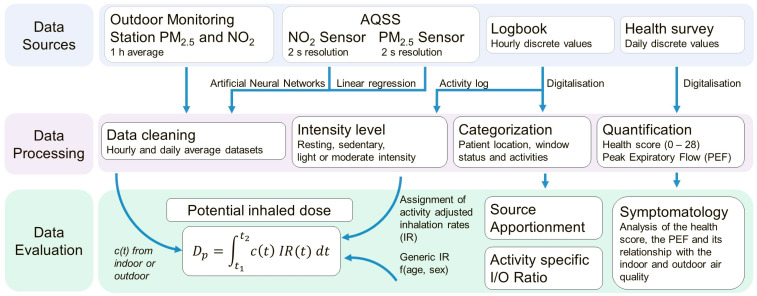
Schema of the data analysis.

As shown in Figure 1, source data from AQSSs, our outdoor monitoring station in Hauptstätter Street, health surveys, and logbooks were merged into a single comprehensive dataset, categorised by date and time, patient ID, and whether the data were collected indoors or outdoors. For the data gathered from participants’ homes, the percentage of completeness after data cleaning was calculated and is presented in Table S5. None of the NO_2_ sensors achieved 100% data completeness due to the exclusion of the warm-up periods.

The raw sensor data were corrected using artificial neural networks (ANN) and linear regression for NO_2_ and PM_2.5_, respectively, followed by conversion into hourly and daily averages. Logbook information was digitalised and categorised into the following groups: patient location, window status, and activities. This information was used in the I/O ratio and the source apportionment studies as well as for the calculation of the activity-specific IR according to the activity intensity levels.

Subsequently, the health score was calculated using the health surveys and, together with the PEF and the AQSS data, evaluated in a qualitative symptomatology study. Finally, the NO_2_ and PM_2.5_ potential doses (*D_p_*) were calculated using pollutant concentrations (*c*(*t*)) from the outdoor monitoring station or the indoor AQSS and the activity-specific IR or the generic IR. The data analysis was carried out in the R software (v 4.2.2). The following sections provide a detailed description of the data processing and evaluation.

#### 2.4.2. Sensor Data Quality Assurance

The calibration and evaluation of the sensors took place before deployment. A separate paper focusing on the calibration and performance evaluation of the NO_2_ and PM_2.5_ sensor units will be published soon. Data for PM_2.5_ concentrations were taken from the raw sensor data every two seconds. First, data from times with any sensor overflow errors were removed. Next, the univariate linear regression shown in Equation (1) was applied to the PM_2.5_ data before hourly averages were taken.

(1) $$c_{corr}=m\;c_{raw}+b$$

Here, $c_{corr}$ is the corrected pollutant concentration in μg m^−3^, *m* is the correction coefficient, $c_{raw}$ is the sensor’s raw reading in μg m^−3^, and *b* is the correction constant or offset in μg m^−3^. These correction factors were found by co-locating reference-grade instruments and the indoor and outdoor AQSSs in indoor and outdoor conditions, respectively. Table S6 lists the values of the correction parameters for each sensor. The outdoor PM_2.5_ sensor had a dryer at its inlet to avoid the effect of hygroscopic growth of particles at high RH. A detailed evaluation of the thermal dryer is described in Chacón-Mateos et al. [40]. 

The corrections of NO_2_ sensor data were achieved using machine learning (ML) methods. Several ML models were investigated to determine which model best corrected the data for variations caused by changes in temperature and RH. Models applied during the study were support vector regressor (SVR), random forest regressor (RFR), and ANN together with multiple linear regression (MLR). Each model was run on both indoor and outdoor data. The predictions of the ML models were compared with the NO_2_ concentrations measured by diffusion tubes placed in participants’ homes alongside the indoor and outdoor AQSSs. ANN results were most comparable to the passive sampling data. Moreover, ANN was deemed to be the most robust, and capable of handling the influence of the RH and temperature on the sensor signal. The NO_2_ sensor data were first averaged in 10 min intervals before being processed using the ANN model.

#### 2.4.3. Activity Specific I/O Ratio

To investigate if AQSSs can be used to determine which indoor activities contribute most to indoor pollution and the effect of outdoor air and ventilation on indoor air, the I/O ratio was calculated for the nine logged activities. For hours during which participants logged multiple activities, each listed activity was considered individually rather than treating the multiple activities as a group. By splitting groups of activities, the calculated hourly concentration is attributed to each of the activities happening in that hour. This method is used to approximate the contribution of each activity to the total pollutant concentration but may lead to estimation errors. Take as an example, two activities logged in an hour, one which generates substantial pollution (cooking) and the other which does not generate any (sleeping). By assigning the hourly pollutant concentration average to the high-emission activity (cooking), its overall contribution may be underestimated. The reverse is true for a low-emission activity (sleeping), in that its contribution to the hour may be overestimated using this method.

To determine the I/O ratio associated with each activity, only indoor activities were included, and only if they occurred for multiple patients in the dataset. The categories “Not Home” and “Garden or Balcony” were therefore excluded from this analysis. For patient 4, who had two outdoor sensors, the data from the street-side sensor were used. As the indoor PM_2.5_ concentration of patient 7 was, on average, 12 times higher than all other participants, the I/O ratio for PM_2.5_ was evaluated separately. The outdoor PM_2.5_ sensor in the house of patient 6 stopped working properly from 19 April 2020 until the end of the deployment on 30 April 2020, and therefore, only the first 15 days were used for the data analysis. Patients 2 and 5 were excluded from these results due to the lack of outdoor sensor data.

#### 2.4.4. Source Apportionment

The activities recorded in the logbooks, combined with the sensor data, were used to determine the variation in indoor air quality due to participant actions. The goal was to determine which activities generated the highest pollution concentrations for NO_2_ and PM_2.5_ and whether they could be tracked using stationary AQSSs. For this analysis, activities were included as they were reported in the logbook, with multiple activities occurring during the same hour. Activities are only included if they occurred for a minimum of 30 h across all patients to focus only on the most common combinations of activities. 

#### 2.4.5. Symptomatology

To assess the feasibility of using AQSSs to investigate the relationship between symptom severity and air quality, a health questionnaire was developed based on the Asthma Control Test (ACT) and COPD Assessment Test (CAT). Symptoms included in the questionnaire were the following: feelings of tightness in their chest, dyspnea or shortness of breath, cough, sputum or coughing up mucus, wheezing, impairment of their daily life, and how much they used their rescue inhaler during the day. A sample of the health survey is shown in Figure S3 in the Supplementary Materials.

A health score was calculated based on the results of the daily questionnaire regarding the presence and severity of symptoms. Each of the possible answers to the questions in the health survey was rated from zero to four. The health score was calculated as the sum of these individual points for each single day. The maximum score possible was 28. A high health score means that the patient’s symptoms were more severe. A score of zero would mean no symptoms were present on the day. 

The results of the daily PEF measurements were analysed without further processing in L min^−1^. The PEF device was able to measure airflows between 50 and 800 L min^−1^. The health scores and the daily PEF measurements were examined individually for each patient.

#### 2.4.6. Exposure Assessment

In this section, the calculations of the potential inhaled dose are explained. First, each activity in the logbook was assigned to an intensity level, based on how strenuous the activity may be. These estimates of intensity are taken from Chapter 6 of the U.S. EPA’s Exposure Factors Handbook [41]. There are five intensity levels used here, as defined by the EPA. Resting corresponds to sleeping or napping, sedentary describes mainly static activities such as watching television or reading, light includes cooking or other standing activities, and moderate is described as walking, easy cycling, or climbing stairs [41]. This classification is used to predict the IR of a patient for the hour. The IR and the intensity level assigned for each activity according to age and sex are shown in Table S7. For hours where no activities were recorded, the generic IR was used. No IR or dose was calculated for the periods when patients were not home as the patient would not be in the same area of the sensor, making the calculations inaccurate for their true exposure.

Given that multiple activities with varying intensities were sometimes recorded within the same hour, we conducted an analysis to assess the influence of IR variability. We calculated the minimum, maximum, and mean IR and used them to estimate the potential inhaled dose indoors. The mean was estimated under the assumption that each recorded activity accounted for equal fractions of the hour. For example, if both “Cooking” and “Sleeping” were marked for 8:00 am, it was assumed that the person cooked and slept for 30 min each. To calculate the hourly minimum (or maximum) IR values, the lowest (or highest) intensity activities were used. The results were compared against the potential dose calculated using a generic IR. For the pollutant concentration, the indoor sensor data were used. Hours for which no specific activity-adjusted IR existed, for example, “Visitor”, were assigned the generic IR. Only days when 85% of activity data were completed were included.

After determining the hourly IR values, the potential inhaled dose *(D_p_*), which is the amount of pollutant that is inhaled by an individual [41], can be calculated as shown in Equation (2) [42].

(2) $$D_{p}=\int_{t_{1}}^{t_{2}}c\left(t\right)\;IR(t)\;dt$$

where *c* is the indoor or outdoor pollutant concentration, *IR* is the inhalation rate, and *t* is the time. Doses are often presented as dose rates, i.e., the amount of dose per unit time (e.g., µg hour^−1^) [42].

Krause [43] proposes three methods for calculating the potential dose. The first is to use a generic IR together with the pollutant data from an outdoor monitoring station. The second is to use the generic IR and pollutant concentrations from a portable AQSS, which is deployed in the same microenvironment as the individual. The third method is to calculate the potential dose from the activity adjusted IR and the personal AQSS pollutant data. In our work, an additional fourth method is used, which is to calculate the dose using the activity-adjusted IR with the outdoor monitoring station data. This fourth method is used to give a complete comparison of exposure estimation variability using local indoor or ambient outdoor data. Additionally, the comparison of the two IR (generic or activity-adjusted) calculations using AQSS data may give insight into the importance of using the activity-adjusted IR. If the calculated doses were similar, it would show that the use of an adjusted IR is unnecessary. In summary, the four methods used to calculate potential inhaled dose are as follows:

(A) Generic IR + outdoor monitoring station data
(B) Activity-adjusted IR + outdoor monitoring station data
(C) Generic IR + indoor AQSS data
(D) Activity-adjusted IR + indoor AQSS data

## 3. Results

### 3.1. Relationship between Indoor and Outdoor Air Quality

#### 3.1.1. General Comparison

**Figure 2:**
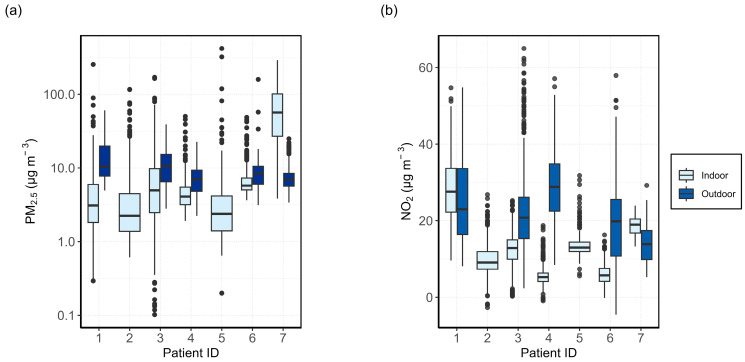
Hourly concentrations of (**a**) PM_2.5_ and (**b**) NO_2_ for each patient over the entire measurement period. Note that the *y*-axis in panel (**a**) is on a logarithmic scale and that, in panel (**b**), the unexpected high NO_2_ concentrations in the house of patient 1 may be due to an overestimation caused by the ANN correction.

To make a comparison of the indoor and outdoor air quality measured by the AQSSs, the hourly concentrations of PM_2.5_ and NO_2_ for the entire measurement period are plotted in Figure 2 for each patient. The central box of the boxplot contains 50% of the data (interquartile range, IQR) and the inner line corresponds to the median. The whiskers extend to the smallest and largest observations within 1.5 times the IQR from the quartiles, with outliers plotted as individual dots. Outdoor data for patient 4 are taken from the street-facing AQSS. Patients 2 and 5 did not have any outdoor data to report. 

The outdoor PM_2.5_ concentrations were higher than the indoor concentrations for patients 1, 3, and 4. Median PM_2.5_ concentrations in the home of patient 6 were similar for both indoor and outdoor microenvironments, with more occasional peaks observed indoors. All patients, excluding patient 7, had roughly similar indoor PM_2.5_ concentrations among them, with median measurements falling below 10 µg m^−3^. Patient 7 had much higher concentrations of PM_2.5_ inside than outside, with the median concentration above 40 µg m^−3^. From the logbook and the environmental questionnaire, it is known that patient 7 often lit multiple scented candles in the house and blew them out without proper ventilation, which is most probably the reason for the high PM_2.5_ concentrations. 

The comparison of indoor and outdoor NO_2_ is shown in Figure 2b. Some slightly negative values were measured for NO_2_ (patients 2, 4, and 6) which is an artefact of the ANN models used to process the data. We should also take into consideration that it is especially difficult to measure low NO_2_ concentrations precisely with the tested electrochemical sensors as the relative expanded uncertainty is considerably larger than for high concentrations [44]. The average concentrations together with the relative expanded uncertainties (REU) of the NO_2_ and PM_2.5_ measurements are presented in Table S8.

For NO_2_, the outdoor concentrations were higher than indoors for patients 3, 4, and 6. Patients 1 and 7 showed the opposite trend. For patient 1, indoor NO_2_ concentrations were roughly 30 µg m^−3^. This is more than twice as high as the indoor NO_2_ measurements of most other patients. As NO_2_ passive samples were not collected for patient 1, it is challenging to determine whether the elevated indoor NO_2_ concentrations accurately reflect true conditions or are a result of the ANN model used for calibration. Previous analysis of ML and MLR correction models does suggest an overestimation of NO_2_ concentration by the ANN model for patient 7 [45].

**Figure 3:**
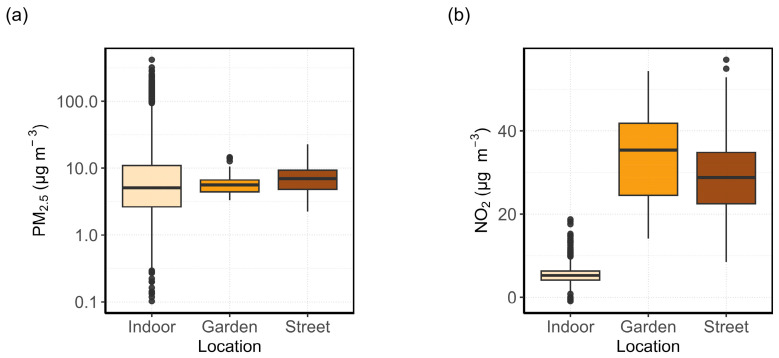
Hourly concentrations of (**a**) PM_2.5_ and (**b**) NO_2_ for patient 4 at indoor, garden, and street locations. Note that the *y*-axis in panel (**a**) is on a logarithmic scale.

For patient 4, two outdoor AQSSs were installed to determine if there were measurable differences in air quality on opposite sides of the house. Figure 3 shows the PM_2.5_ and NO_2_ concentrations measured indoors, as well as outside on the garden and street sides of the house. Both PM_2.5_ and NO_2_ concentrations are lower indoors than outdoors. In general, PM_2.5_ concentrations tend to be more variable indoors compared with outdoors, while the opposite trend is observed for NO_2_, with more consistent levels indoors and greater variability outdoors.

The street-facing AQSS measured higher PM_2.5_ concentrations than the unit placed in the garden. There was a larger spread in garden-side NO_2_ measurements, with a median of 35 µg m^−3^, slightly higher than for the street-facing side (29 µg m^−3^). The REU for the garden-side NO_2_ sensor at the median was ±13 µg m^−3^ and was greater than the REU of the street-side NO_2_ sensor (±7 µg m^−3^). It is possible that the overestimations of the NO_2_ concentrations seen at the garden side were due to the ANN calibration. The NO_2_ passive samples measured an average of 24 µg m^−3^ and 23 µg m^−3^ for the street and the garden side, respectively, during the same period. This may indicate that the street sensor slightly overestimated the NO_2_ concentrations, whereas the sensor placed in the garden overestimated the NO_2_ concentrations by approximately 12 µg m^−3^.

**Figure 4:**
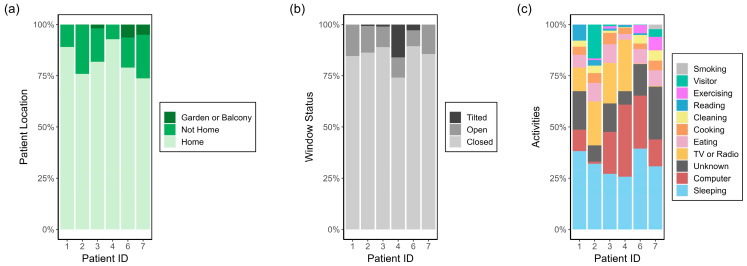
Percentage of patients’ time grouped by (**a**) patient location, (**b**) window status, and (**c**) activities at home.

The percentage of time that each patient spent at home, the status of the windows in the living room, and the time contribution of home activities are visualised in Figure 4. All patients spent the majority of the time at home, 83% on average, which is consistent with the results of previous studies [24]. Patient 2 spent the least amount of time at home (76%) while patient 4 spent the most time at home (93%). This means that the home environment is a crucial part of understanding personal exposure. The percentage of time spent at home in our study is slightly higher than the statistics compiled by Klepeis et al. [10], which reported that most people are at home for roughly 70% of the day. This difference could be due to COVID-19 related restrictions happening from the middle of March 2020 and lasting until the end of April 2020. The COVID-19 restrictions affected patients 3, 4, and 6.

All patients had the windows closed for the majority of the time, 85% on average, with only four patients ever reporting to have tilted the windows, as opposed to fully opening them. Given that the measurements took place in winter and spring, it is expected that the windows would be closed most of the time. The activities that patients spent the most time on were “Sleeping”, “Computer”, and “TV or Radio”. “Unknown” also accounted for a significant amount of each patient’s time.

#### 3.1.2. Activity Specific I/O Ratio

**Figure 5:**
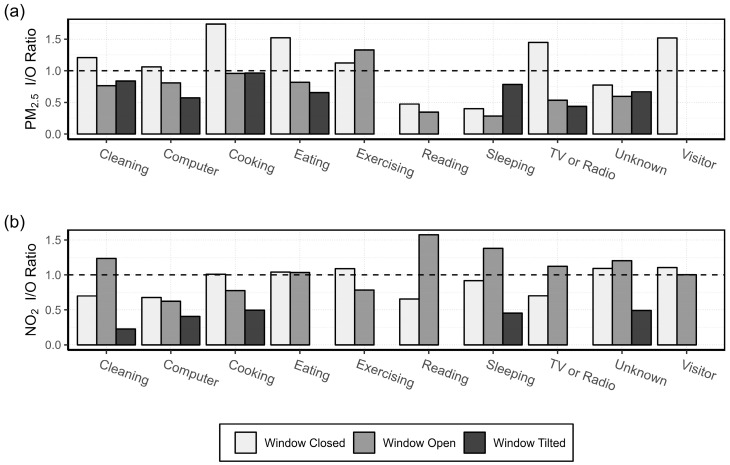
I/O ratios for (**a**) PM_2.5_ and (**b**) NO_2_ associated with individual activities, grouped by window status.

The mean I/O ratios associated with each activity, under various ventilation conditions (window closed, open, or tilted), are presented in Figure 5 for four of the participants (for patients 2 and 5, outdoor AQSS data were not available). As the PM_2.5_ data collected in the house of patient 7 were extremely high due to the scented candles, the results of the activity specific PM_2.5_ I/O ratio of patient 7 are presented in Figure S4. 

For all activities, PM_2.5_ had the largest I/O ratio when the windows were closed, indicating that there are significant sources of PM_2.5_ indoors. Another factor that may influence the results is the measurement period. The measurement campaign was conducted during the colder months when ventilation is typically minimised to reduce energy costs. Without adequate ventilation, the generated PM_2.5_ can stay several hours in the air. The activities “Cleaning”, “Cooking”, “Eating”, “TV or Radio”, “Computer”, “Exercising”, and “Visitor” were found to be significant indoor sources of PM_2.5_ when the windows were closed. Only “Sleeping” and “Reading” had an I/O ratio of less than one for all window statuses, indicating that the PM_2.5_ concentration outdoors was higher than indoors. A fact observed in Figure 5 is that opening or tilting windows effectively reduces indoor PM_2.5_ concentrations during activities such as “Cleaning”, “Computer”, “Cooking”, “Eating”, and “TV or Radio”. The only activity that showed the opposite trend was sleeping. This is because indoor PM_2.5_ concentrations were lower than outdoor concentrations during sleeping; therefore, keeping the window tilted for the whole night increased the I/O ratio. A detailed analysis of the hourly indoor and outdoor PM_2.5_ and NO_2_ concentrations together with the activities and the window status can be seen in the Supplementary Materials, Figures S5–S26. The results are presented in one-week segments, to be able to better associate pollutant peaks with specific activities and window status.

The I/O ratios for NO_2_ were lower than one during most activities, suggesting there were fewer sources of NO_2_ indoors. When the windows were closed, the only activities with an I/O ratio slightly greater than one were “Cooking”, “Eating”, “Exercising”, “Unknown”, and “Visitor”. Cooking is known to be a source of NO_2_, especially from the use of gas stoves or ovens [46]. Eating and cooking were frequently marked at the same hour. Moreover, the concentrations measured during “Eating” may have been created in the previous “Cooking” activity and what we measured was the “Post-cooking”, i.e., the transportation of pollutants from the kitchen to the living room where the AQSS was located. For 50% of the activities, i.e., “Cleaning, “Reading”, “Sleeping”, “Unknown”, and “TV or Radio”, the I/O ratios for NO_2_ were higher than one when windows were open. This is in accordance with findings from Stamp et al. [47], who recorded increased I/O ratios for NO_2_ when windows were open. Other activities such as “Computer”, “Cooking”, and “Exercising” showed higher NO_2_ I/O ratios when the windows were closed or tilted as compared with when they were open. 

#### 3.1.3. PM Advisory Study

**Figure 6:**
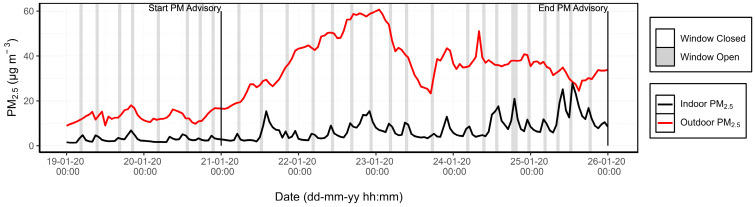
Indoor and outdoor PM_2.5_ concentrations measured by the AQSSs deployed at the home of patient 1 during the PM alert, along with the corresponding window status. Periods when the window was open are indicated by a grey background.

From 21 January to 26 January 2020, a PM advisory (*Feinstaubalarm*) was active in Stuttgart during which sensors were deployed at the home of patient 1. These alerts are issued in conjunction with the German Weather Service (DWD) when certain conditions are met, including PM_10_ levels exceeding 30 µg m^−3^, the absence of rain, and low wind speeds. Figure 6 shows the hourly PM_2.5_ concentrations from the indoor and outdoor AQSSs during the event and the two preceding days. It can be observed that the outdoor PM_2.5_ concentration reached its maximum (60 μg m^−3^) on the night of 23 January and that the average PM_2.5_ concentrations measured indoors increased gradually throughout the entire period of the PM alert.

**Figure 7:**
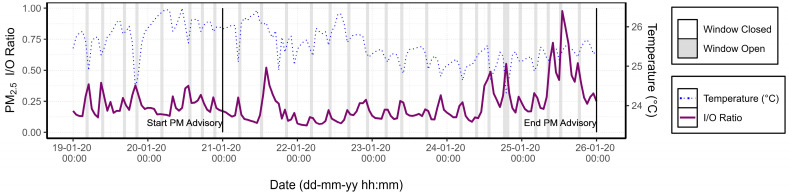
Time series of the mean hourly I/O ratio for PM_2.5_ and indoor temperature during the PM alert, combined with window status.

The I/O ratio was analysed alongside window status and temperature to assess the impact of ventilation on indoor air quality under poor outdoor air quality conditions, as presented in Figure 7. A marked difference was observed in the change of the PM_2.5_ I/O ratio when the windows were open. The peaks represent increases in indoor PM_2.5_ concentrations as polluted outdoor air enters through the open window. In the six days preceding the PM alert, the average I/O ratio increased by 16% when the windows were open. However, during the PM alert period, the I/O ratio rose by 49% when the windows were open, compared with when they were closed. This suggests a significant impact of window opening on indoor air quality during periods of elevated outdoor pollution. Temperature drops on the 20th and 23rd, which were not associated with open windows, coincided with peaks in the I/O ratio. This confirms that the windows were open during those hours even though the patient did not register it.

Moreover, Figure 7 evidences that the PM_2.5_ I/O ratio during nighttime hours progressively increased each day throughout the PM advisory period. This trend can be attributed to the infiltration of outdoor air into the house, which can occur through structural imperfections in window seals or doorways [48]. This phenomenon was observed in the home of patient 1, who reported in the environmental survey that the windows were 40 years old and inadequately sealed. Although the windows were kept open 2% less frequently during the PM alert period compared with the preceding week (16% vs. 18% of the time), indoor PM_2.5_ concentrations increased by 85% during the alert period. 

**Figure 8:**
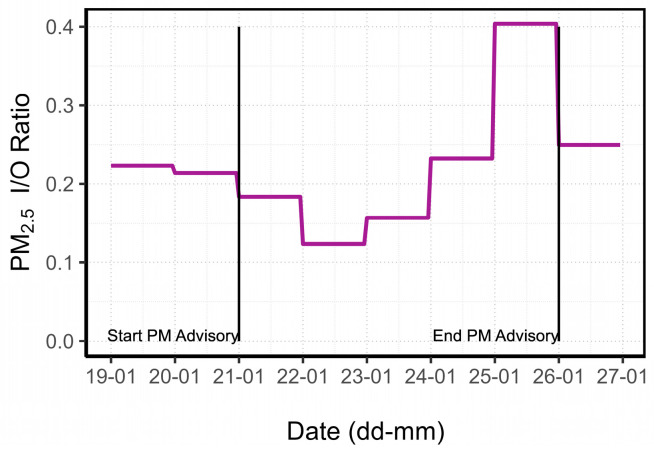
Daily PM_2.5_ I/O ratio before, during, and after the PM alert.

The daily mean of the I/O ratio during the PM advisory and the two preceding days is shown in Figure 8. The daily indoor and outdoor PM_2.5_ concentrations can be seen in Figure S27. There was an increase in the I/O ratio from 22 January to 25 January before it decreased again. The trend of increasing daily I/O ratio, together with the progressive rise in indoor PM_2.5_ concentrations, may prove the infiltration of PM_2.5_ in the home. The outdoor PM_2.5_ concentrations began to decrease gradually on the 23rd, but the I/O ratio did not decrease until the 26th.

### 3.2. Source Apportionment

**Figure 9:**
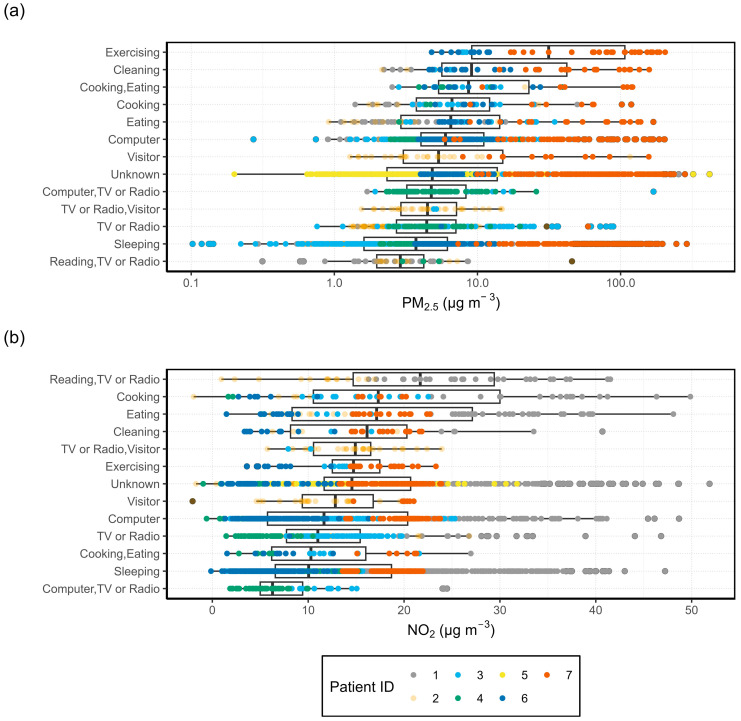
Indoor (**a**) PM_2.5_ and (**b**) NO_2_ concentrations associated with an activity or group of activities; patient values represented by colour-coded points. Note that the *x*-axis in panel (**a**) is on a logarithmic scale.

The results of the PM_2.5_ and NO_2_ concentrations associated with each activity are shown in Figure 9. Here, activity and pollutant data were grouped across multiple patients. The individual results of all patients, except for patient 5 due to lack of activity data, are presented in the Supplementary Materials in Figures S28 and S29.

The highest NO_2_ values across all activities were measured in the house of patient 1. As explained in Section 3.1.1, the ANN model used for the calibration of the sensor may have overestimated the concentrations in this particular case. The highest NO_2_ concentrations corresponded to the category “Reading, TV or Radio”. This unexpected result was significantly influenced by the generally higher concentrations measured in the house of patient 1. Other activities that were associated with high NO_2_ concentrations were “Cooking”, “Eating”, and “Cleaning”. Even though most patients had electric stoves, it is also possible for elevated NO_2_ concentrations to occur if the oven is used [46]. Patient 3 was the only participant who used a gas stove, yet they did not exhibit significantly higher NO_2_ concentrations compared with the other patients. NO_2_ is a reactive gas and “deposition and reactions may occur during transport” which may lead to a decrease in concentration over a short distance [46]. This may lead to variations in NO_2_ concentration throughout the home.

As for PM_2.5_, “Cooking, Eating” showed higher concentrations than “Cooking”. Several possible factors may explain the higher concentrations measured during “Cooking, Eating”. Since most patients had closed kitchens, it is possible that emissions were contained within the kitchen until cooking was finished. At that point, the participant would have opened the door to move to another area of the house, allowing the kitchen pollution to reach the living room where the sensor was located. Another possible explanation is, again, the discrete nature of the logbook. It is possible that cooking occurred at the end of one hour, with the majority of any heating steps occurring at the beginning of the next hour and eating occurring at the end. In those cases, the activity group “Cooking, Eating” would contain the times when the most pollutants were generated. For both PM_2.5_ and NO_2_, high concentrations were measured during the activity “Eating”, which may be explained by the same reasons as “Cooking, Eating”. 

As expected, most sedentary or resting activities were not associated with high pollutant concentrations. Concentrations of both pollutants were low during “Sleeping”, “Computer”, and “TV or Radio”. During sedentary activities, PM settles, as it is not agitated by the physical movement of the participants. These hours were shown to have the lowest PM_2.5_ concentrations, which is supported by other studies [43,49].

The activities recorded by Krause [43] differ from those logged in our work due to the use of portable sensors, which allowed Krause the study of different mobility patterns (car, walk, cycle, train, etc.). In their study, all home activities were grouped into one single category, except for sleeping. Though it was not possible to parse out the concentrations during different home activities, their observations during sleep were in good agreement with the results of this study. In both cases, the median PM_2.5_ concentrations fell below 5 µg m^−3^. The results for NO_2_ varied slightly: in Krause’s study, the median was less than 5 µg m^−3^, compared with approximately 10 µg m^−3^ shown in Figure 9.

In Figure 10, the time series of the PM_2.5_ concentration measured in the house of patient 2 is plotted to observe the temporal variation in indoor air quality with respect to the recorded activities. Results for all patients including the window status are shown in the Supplementary Materials, Figures S5–S26.

The time series clearly shows that the PM_2.5_ concentrations reached a minimum during sleeping times (light blue) and increased again in the morning as other actions started to occur. Some peaks, which are marked with arrows, occurred when cleaning or cooking were logged. Hours, when multiple activities were logged, are shown by overlapping colours as seen on 23 January. Other a priori unexpected peaks were also recorded, such as on 21 January, when a PM_2.5_ peak occurred while the patient had marked “Not Home”. This could be explained due to either a logging error by the patient or the presence of another person in the house.

### 3.3. Symptomatology

In this section, the data of the health score and the PEF measurements are combined with the air quality data measured by the AQSSs. The results of the health scores and PEF measurements are given in Table 2. Patients 1 and 3, who were diagnosed with COPD, had higher minimum, mean, and maximum health scores than the other patients, who had asthma. This metric shows that they did have more difficulty breathing in their day-to-day lives. Patients 4, 6, and 7 all reported days when they experienced no symptoms (health score = 0). Patient 5 did not complete the health survey, and patient 2 developed bronchitis during the second week of measurements, necessitating antibiotic treatment. Consequently, their health data have been excluded from the analysis. Missing values in PEF measurements were caused by a delay in the delivery of the peak flow meters.

**Figure 11:**
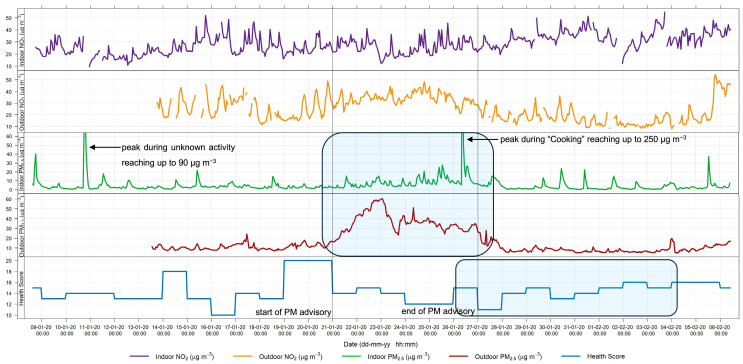
Hourly indoor and outdoor PM_2.5_ and NO_2_ concentrations and self-reported daily health score data from patient 1.

**Figure 12:**
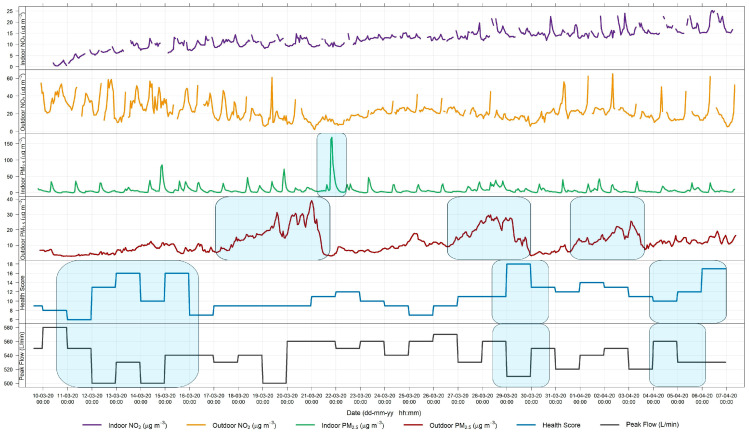
Hourly measured pollutant concentration and self-reported daily health score and PEF data from patient 3.

The qualitative relationship between symptom severity and pollutant concentrations can be observed by plotting the pollutant concentrations and health symptoms together throughout the measurement period for each patient. The results for patients 1 and 3 are shown in Figure 11 and Figure 12, respectively. Events or changes in the severity of symptoms are highlighted with blue rectangles. The results for the remaining patients are presented in the Supplementary Materials (Figures S30–S32).

As discussed in the previous section, there was a PM advisory raised from 21 January to 26 January during the measurements in the house of patient 1. During this time, outdoor PM_2.5_ concentrations increased gradually, and indoor concentrations progressively rose. There were constant changes in the health scores during the month, with the worst scores occurring on the 19th and 20th. During the week following the PM advisory (28 January–5 February), the health score also gradually increased. 

Patient 3, shown in Figure 12, showed several indoor peaks of PM_2.5_ during the measurements, as well as three weeks with increased outdoor concentrations. PEF measurements varied from day to day but consistently decreased whenever the health score increased, indicating that the health score is an accurate method for describing actual patient health. However, this correlation between health score and peak flow varied between the participants. The health score of patient 3 exhibited day-to-day variability. Generally, an increase in the health score was observed some days after a rise in outdoor PM_2.5_. For instance, following the days with elevated PM concentrations (27–28 March), the increase in the health score on 29 March was also accompanied by a decrease in the PEF. A longer study would be necessary to determine if the observed pattern persists over longer periods.

### 3.4. Exposure Assessment

The analysis of personal exposure was carried out in three parts. First, the influence of the estimated activity-adjusted IR on the potential inhaled dose was analysed. Second, the exposure misclassification was examined by comparing data from the outdoor monitoring station or generic IR with indoor data from AQSSs and activity-adjusted IR. Third, the activities with the greatest impact on personal exposure were identified. 

#### 3.4.1. Analysis of the Variability in the Inhalation Rate and Its Effect on the Potential Inhaled Dose

**Figure 13:**
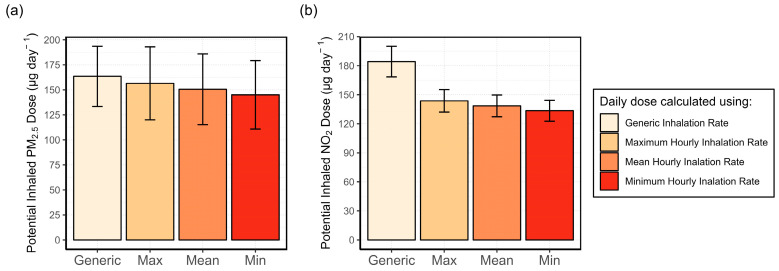
Comparison of daily mean potential inhaled dose of (**a**) PM_2.5_ and (**b**) NO_2_, calculated using the generic IR and the maximum, the mean, and the minimum activity adjusted IR. Error bars indicate the standard deviation in daily dose estimations.

The statistical results of the average, minimum, and maximum daily potential inhaled doses (*D_p_*) of PM_2.5_ and NO_2_, calculated using the activity-adjusted IR, as well as the daily doses calculated using the generic IR, are shown in Figure 13. There is little difference among the maximum, mean, or minimum of the *D_p_* calculated using activity-adjusted IR. This may suggest that hours containing multiple activities do not significantly impact the total daily potential dose. It is also true that, for most hours during which two activities were performed, the activities had the same intensity level, resulting in the same IR. This can be seen in the activities listed in Figure 9 where only the most common activities were included. The most common pairs of activities were “Computer, TV or Radio”, “Reading, TV or Radio”, and “Cooking, Eating”. All activities in these groups are classified as sedentary activities, except cooking which is classified as light intensity. As there is no significant difference between the maximum, mean, and minimum, the mean activity-adjusted IR was used for the calculation of the potential inhaled NO_2_ and PM_2.5_ doses. Moreover, it can also be seen in Figure 13 that the generic IR overestimates the potential inhaled doses compared with the activity-adjusted IR.

**Figure 14:**
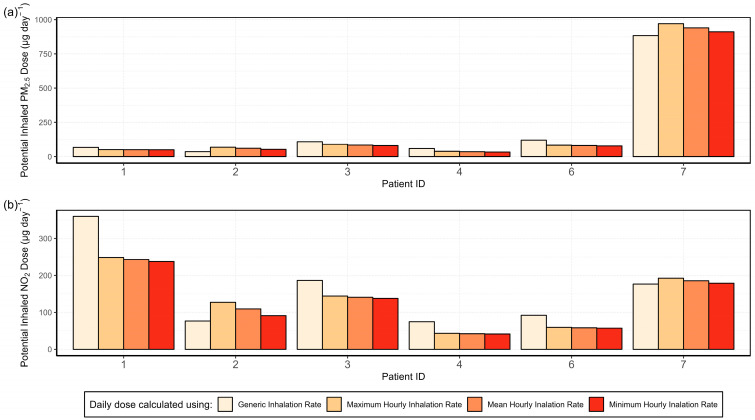
Daily potential dose of (**a**) PM_2.5_ and (**b**) NO_2_, calculated using generic, maximum, mean, and minimum IR, for individual patients.

A comparison of the daily doses for individual patients is shown in Figure 14. Patient 7 experiences up to 5 times higher daily doses of PM_2.5_, due to the habit of lighting scented candles. Patients 1, 3, 4, and 6 show the same pattern as in Figure 13, i.e., generic IR produces the highest dose estimates. However, patients 2 and 7 show the opposite. This is because the calculated daily dose is affected by the individual’s activity level. If an individual is more active at home, their activity adjusted IR will be higher than the generic rate for most of the hours. These differences are shown in Table 3, which lists the mean hourly activity adjusted and generic IR of each patient during active indoor hours, i.e., excluding sleeping hours. Patients 2 and 7 show a higher mean hourly activity adjusted IR compared with the generic IR. In these cases, the exposure will be underestimated when using a generic IR. For all the other cases, the generic IR is overestimated which triggers the overestimation of the daily doses seen in Figure 14. Based on these results, the use of a generic IR for the time spent indoors is not recommended. Further studies including more participants may see more differences in exposure between people with different lifestyles and levels of activity.

#### 3.4.2. Exposure Misclassification

**Figure 15:**
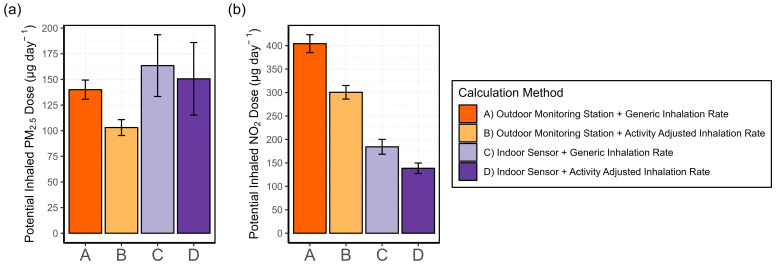
Comparisons of four potential inhaled dose calculation methods for (**a**) PM_2.5_ and (**b**) NO_2_. Error bars indicate standard deviation.

The result of the daily mean potential NO_2_ and PM_2.5_ doses calculated using four different methods (see Section 2.4.6) across all patients is shown in Figure 15. Two trends emerge across the four methods. First, calculating daily potential doses using the outdoor monitoring station data, as in methods A and B, yields significantly different results compared with using indoor data from stationary AQSSs. Second, the use of the generic IR (methods A and C) overestimates the daily dose compared with the activity-adjusted IR.

From Figure 15a, we can also derive that the indoor environment and the individual’s habits have a strong impact on personal exposure. Patient 7 had a median indoor concentration of 56 μg m^−3^, compared with 7 μg m^−3^ at the Hauptstätter Street outdoor monitoring station, due to the continuous use of scented candles and improper ventilation. Thus, the daily potential PM_2.5_ doses calculated including the indoor PM_2.5_ sensor data of patient 7 in the average are much higher than when using the data from the outdoor monitoring station. This indicates that the use of outdoor PM_2.5_ data may result in either over- or underestimation, depending on the individual’s habits. For NO_2_, the trends shown in Figure 15b indicate a clear overestimation of the potential dose when using outdoor data from the monitoring station and generic IR compared with using indoor data and activity-adjusted IR. These findings may extend to other indoor environments with similar conditions and cooking habits. 

The results of the potential dose have been compared with the outcomes from Krause [43] in the AIRLESS project with portable sensors. There, participants were split between those living in an urban area, Beijing, and a peri-urban area, Pinggu. From the comparison of the outcomes, it can be concluded that both studies agree on the fact that using the outdoor pollutant concentration from outdoor monitoring stations may trigger exposure misclassification. 

Krause estimated the exposure in Beijing while at home using the activity adjusted IR and portable AQSSs to be roughly 400 and 300 µg day^−1^ for PM_2.5_ and NO_2_, respectively. Those outcomes are higher than the estimations of 150 µg day^−1^ for PM_2.5_ and 140 µg day^−1^ for NO_2_ using stationary indoor AQSS data and activity-adjusted IR, as shown in Figure 15. The higher potential doses calculated by Krause for AIRLESS study participants may be due to two reasons. Firstly, Beijing has overall higher concentrations of both PM_2.5_ and NO_2_. Median indoor concentrations of PM_2.5_ were nearly five times higher in Beijing compared with Stuttgart (25 versus 5 µg m^−3^). For NO_2_, median home concentrations were roughly 15 µg m^−3^ in the AIRLESS project and 12 µg m^−3^ averaged across participants in this study. The second reason may lie in Krause’s use of a generic IR for the times when participants were at home. As we have seen in Figure 15, the use of a generic IR overestimates the results of potential PM_2.5_ and NO_2_ doses. In our study, we have proven that “home” is a very complex microenvironment where multiple activities with different intensity levels can occur. Considering that, in both studies, patients are at home for more than 80% of the time, using a generic IR for the entire time spent indoors may also be a source of exposure misclassification. 

#### 3.4.3. Activity-Specific Potential Inhaled Dose

**Figure 16:**
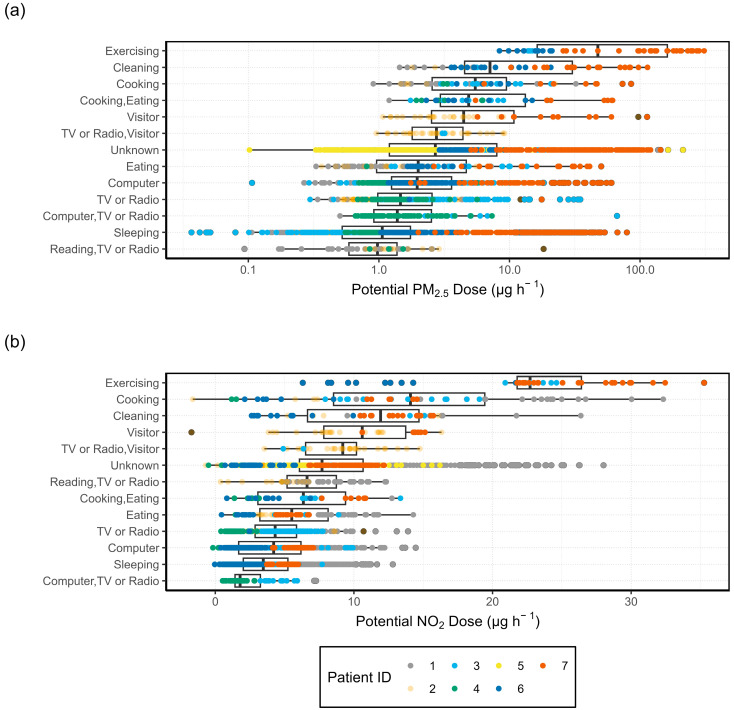
(**a**) PM_2.5_ and (**b**) NO_2_ potential inhaled dose associated with each unique group of activities. Patient values represented by colour-coded points. Note that the *x*-axis in (**a**) is on a logarithmic scale.

To identify the indoor activities contributing most to personal exposure, the potential dose for PM_2.5_ and NO_2_ was calculated for each hour and grouped by the activities occurring during that hour, as shown in Figure 16. It is clear from Figure 16 that the highest potential doses were recorded during the most strenuous activities. “Exercising” was the activity with the highest intensity from those recorded on the activity log and is associated with the highest hourly doses of both PM_2.5_ and NO_2_. For PM_2.5_, the median dose during “Exercising” (about 16 µg hour^−1^) is more than twice as much as the next highest potential dose which occurs during “Cleaning” (about 6 µg hour^−1^).

Calculating the potential dose using an activity-adjusted IR changes the weight that each activity carries for personal exposure as compared with simply using a generic IR. When looking at exposure based on potential dose, more strenuous activities such as exercising contribute more than sedentary ones. Activities such as cooking or cleaning, which are both known to generate pollutants and fall into the “light” or “moderate” intensity level, have both the highest concentrations and potential doses. Given that different indoor activities contribute variably to personal exposure based on their associated concentrations and/or intensity levels, it is important not to overlook these variations by relying on a generic IR for the time at home.

## 4. Discussion

### 4.1. Air Quality Sensors

#### 4.1.1. Data Loss and Data Quality

The percentage of completeness of the sensor data was between 92% and 100% for the PM_2.5_ sensors and between 76 and 99% for the NO_2_ sensors. Data losses could have been reduced by adding Wi-Fi or LTE modules to the sensor systems. In that way, misfunctions and problems could have been identified sooner. The exclusion of data from the warm-up period for the NO_2_ sensors caused the loss of the first hours in each of the sensor deployments, from 4 up to 24 h.

The primary challenge regarding the uncertainty of the sensor data lies in determining the suitability of the calibration parameters obtained two weeks prior to the campaign for use during the measurements at homes lacking reference instruments, particularly for indoor microenvironments. The transfer of calibration parameters becomes complex when relocating sensors from their original co-located positions, and ensuring accurate performance in the new location cannot be guaranteed [50]. The use of passive samples for NO_2_ is a simple tool to obtain a reference value. 

During the study, we detected inconsistencies in some of the calibrated NO_2_ sensor data, for instance, the higher NO_2_ concentration in the garden side compared with the street side during the measurements at the home of patient 4. Moreover, the indoor NO_2_ concentrations at the home of patient 1 were most probably overestimated too. However, without a reference value, it is not possible to validate these results. Future studies should explore effective strategies for managing calibration transfer. 

Lastly, some negative values were still present in the NO_2_ datasets after calibration (patients 2, 4, and 6). Measuring low concentration levels with the current NO_2_ electrochemical sensors implies a higher uncertainty of the measurements. However, we have seen that concentration peaks are well detected. Considering this, air quality sensors have more potential in those regions or indoor microenvironments where high concentrations are expected.

#### 4.1.2. Use of Stationary Sensors

The AQSSs were positioned in the living room, chosen based on participants’ reported highest occupancy, to ensure monitoring in the area most frequented by the patients. There is a possibility of underestimating exposure during activities occurring outside of the living room. Questions were raised, especially regarding cooking, which is known to raise pollutant concentrations. This may be particularly important for patients who have closed kitchens. In the future, multiple sensors could be placed in multiple areas of a single home to determine the difference across several rooms. Consideration should be given to deploying sensors in additional indoor environments, such as workplace offices, where patients routinely spend significant time. This broader sensor placement strategy can provide a more comprehensive understanding of personal exposure patterns.

The stationary deployment of AQSSs raises concerns about the accuracy of personal exposure estimates, particularly when patients are not in the sensor’s vicinity, such as when away from home. However, the convenience and minimal disruption afforded by stationary AQSSs in the home, as opposed to carrying portable sensors, are advantageous, especially for long-term studies.

The results of this study, using sensor systems at fixed locations, were compared with those of a similar study from Krause [43] using portable sensors. During the study with portable sensors, it was found that even though the highest dose per minute was observed during commuting, the time in transit accounts for less than 10% of total time, with the home environment emerging as the predominant contributor to the total dose [43]. This suggests that our methodology of using stationary sensors for indoor and outdoor microenvironments may be sufficient to estimate exposure in long-term studies but may lack the sensitivity required to investigate short-term health effects resulting from pollution peaks.

Although it was shown that the exposure estimates calculated using data from indoor stationary sensors and portable sensors were similar due to the predominant contribution of the home environment, there remain scenarios where stationary sensor data may not suffice for determining exposure. In our study, the patients spent an average of 83% of their time at home. Individuals who spend more time outside their homes may necessitate the use of portable sensors or multiple stationary sensors strategically placed in environments where they spend the most time. This consideration is particularly crucial in regions with high pollution levels where outdoor exposure may significantly impact overall exposure levels. 

### 4.2. Nature of Participant-Reported Data

Assessing the veracity of self-reported data from participants poses a significant challenge. In certain instances, recorded parameters may be correlated with proxy variables that facilitate the validation of reported activities. For example, sudden temperature changes may be an indicator of the opening of a window. However, most other activities recorded by patients are more difficult to verify. 

Accurately determining individuals’ actions without invasive monitoring presents a complex challenge. However, invasive monitoring may not always be feasible due to participant reluctance or study demands. Participants may be unwilling to continuously wear sensors or record data for extended periods. If study requirements are overly demanding, there is a risk of non-compliance. Providing monetary compensation to volunteers is an option, but it does not guarantee data quality.

While there is no feasible method to ensure complete cooperation and accuracy in recording data, good communication with participants, education about the study’s objectives, proper training, and regular checks can enhance the likelihood of complete and accurate data collection. 

### 4.3. Activity Specific I/O Ratio 

The I/O ratios of activities, when grouped by window status, were affected by the discrete nature of activity reporting. Patients could only record an event as occurring for a full hour. Especially in winter, it is unlikely that any patient kept the windows open for an entire hour. Additionally, there could be a lag between certain activities and the measurement of the pollutant, resulting in peak concentrations being recorded in the following hour and causing the peak to be misassigned. Diapouli et al. [51] found that in their study of the I/O ratio of PM mass and number concentration in residential buildings during various activities, cooking caused the highest I/O ratio, which is consistent with the results for PM_2.5_ presented in Figure 5. Together with “Cooking”, hours when “Eating” occurred had high I/O ratios as well. “Eating” often took place in the same hour as, or immediately following, cooking. Most patients had closed kitchens, which could result in pollutants not reaching the sensors in the living room until cooking was finished and the patient had moved to another room to eat. These results include all instances of “Eating”, whether it was the only activity during the hour or not. If “Eating” occurred simultaneously with “Cooking”, the I/O ratios may be overestimated.

### 4.4. Source Apportionment

The results of the source apportionment showed that the data of the stationary PM_2.5_ and NO_2_ sensors together with the logbook are able to accurately attribute peaks in concentration to indoor activities occurring in the same hour. For this analysis, the activities that were recorded together for the same hour were considered as a unique group. In essence, “Cooking” is treated as a separate activity from “Cooking, Eating”. When it was distributed to patients, 13 single activities were listed on the activity log. After completion of the pilot study, 113 unique groups of activities were recorded among all patients. This emphasizes the difficulty of discretizing the contribution of any single activity to personal exposure. It is not possible with this method to isolate emissions from a single activity during hours when multiple activities were logged simultaneously. Still, from the methods used here, general conclusions can be made about which indoor activities generate the highest pollutant concentrations.

### 4.5. Symptomatology

There is uncertainty in health data because symptoms vary greatly among individuals. Many factors can trigger asthma symptoms, including weather, exposure to allergens or other irritants, activity level, and strong emotions [52]. These triggers may build on each other to further exacerbate symptoms. This complexity makes it challenging to discern which symptoms are directly related to changes in air quality.

Moreover, the differences in how individuals perceive their symptoms make comparisons between multiple participants more complex. To overcome that, the health questionnaire was designed specifically to avoid the subjectivity of the patients, with answers aiming to quantify the symptom severity (e.g., coughed once briefly, coughed briefly several times, coughed almost every hour, etc.). Overall, the trends in how symptoms change become more important in drawing conclusions about larger populations. An increase in symptom severity is a strong indicator of how one’s health is affected.

The use of other quantitative measurements can have disadvantages when participants complete measurements themselves. Even a simple lung function test may pose compliance difficulties in longer-term studies. Firstly, the training of patients by qualified personnel is crucial for correct data collection. Secondly, the effort a single patient puts in can vary from day to day, impacting the reliability of the results. It is known that patient motivation drops with time. Jiang et al. [53] found that in a two-week study of asthma symptom tracking, patient compliance had already dropped by the second week. Another study on asthma patient compliance with long-term PEF measurements found that after 6 months, only 50% of participants still recorded accurate PEF values [54]. Addressing these challenges is crucial for ensuring the reliability and validity of the data collected over extended periods.

Despite the limitations of patient-recorded health data, there are several advantages of using a symptom log. First, it is one of the least invasive methods for obtaining health data, as patients do not need to visit a medical office or make appointments. Secondly, the only type of lung function testing undertaken was the PEF, which is simple and easy to do at home. The health data collected in this study was independent of the day of the week, as patients reported daily. Additionally, unlike studies using hospital admission data, symptoms were recorded across all levels of severity. This enabled the identification of the onset of symptom aggravation, rather than solely noting when symptoms reached a specific severity threshold. Future studies with a large number of participants could investigate the lag effect between changes in symptoms, including the evaluation of specific symptoms, and changes in pollutant concentrations. Such studies could examine correlations starting from the same day and extending up to a week later, as identified to be the longest delayed response time [55].

In summary, although this pilot study cannot determine the precise influence and lag between peaks in pollutant concentration and subsequent increases in health scores, the presented results demonstrate the potential use of AQSSs for environmental epidemiology at fixed locations. Stationary sensors collect data from indoors and the surroundings effortlessly for the patient, which makes them an appropriate technique for long-term epidemiological studies. The continuous indoor and outdoor monitoring gives a better understanding of the quality of the air each individual breathes. 

Moreover, the health score system developed for this study consistently shows plausible information on symptom intensity with fluctuations from day to day, so that changes in the state of health or symptom burden are easy to understand and can be analysed using the point scores. Even with varying disease severity, there are sufficient intra-individual fluctuations to document day-dependent deviations in symptom burden and relate them to the air quality data. The PEF measurement is a useful addition as an objective parameter.

### 4.6. Exposure Assessment

One of the current limitations of determining personal exposure is the variability in individual behaviour and activities. In this study, the activity log was used to determine the patient’s activity level. However, there are some challenges when using a method such as the activity log. Partly, this is due to the previously mentioned limitations of using participant self-recorded data. However, some difficulties occur when participants must select actions for a discrete amount of time. For instance, there were many cases during the study where participants had marked multiple activities in one hour. This is logical, given that not all activities last a full hour, or start exactly on the hour. This practice introduces uncertainty, particularly when activities vary in intensity, potentially affecting the accuracy of exposure estimates. In this study, the mean IR was used, assuming equal time allocation for each recorded activity within an hour. The results of the variability analysis indicated that this assumption did not significantly alter the estimated exposure outcomes.

Another potential source of uncertainty is the methodology to calculate the potential inhaled dose. In this study, each activity was considered separately, and the entire amount of pollutant measured during an hour was attributed to each activity performed in that hour. This approach may have led to the under- or overestimation of the potential dose associated with each activity. For instance, if a “sedentary” activity such as sleeping occurred in the same hour as a “light” activity with high emissions like cooking, the dose attributed to the sedentary activity would be overestimated due to the contribution from the more active one. 

The variation in the IR, which depends on overall individual fitness or health, may contribute to uncertainty in personal exposure estimates. IR values predicted by the EPA are typically calculated for healthy populations [56]. In this study, no adjustments were made to account for the fact that participants had respiratory problems. However, evidence suggests that this may not significantly affect accuracy. Corlin et al. [56] studied the IR in a population with a high percentage of individuals with respiratory or cardiac health conditions and found that EPA estimates remained accurate. They included adjustments for weight in their calculations, which were not possible in our study. Given the limited number of studies addressing this concern, it should be considered in future research.

Another limitation of using the EPA-estimated IR is the somewhat loose definition of activity intensity. For instance, if a patient recorded “Exercising”, it was assumed to be light exercise indoors and labelled as “moderate intensity”. However, the actual intensity could vary, either higher or lower, without confirmation of the exact nature of the exercise. For future studies, recording and including heart rate data could enhance the accuracy of determining an individual’s activity level and improve IR calculations. Additionally, investigating the duration of elevated IR during more intense activities would provide valuable insights.

Finally, the comparison of the results of the potential inhaled dose with the AIRLESS study has shown that (I) both studies agree on pointing out outdoor air quality data from outdoor monitoring stations as a possible source of exposure misclassification, and (II) the results may be overestimated when assuming a generic IR for the time spent at home. It would be worth considering the possibility of assuming a generic IR for the time participants spend outdoors rather than for the time they spend indoors. As nearly 80% of the exposure occurs in the home environment, the contribution of travel methods (car/bus, train, walk, cycle, motorcycle) represents a small percentage of the total dose. This approach could reduce the exposure misclassification caused by the use of generic IR for the time spent at home while minimizing participant effort. In regions where people spend most of their time indoors, it may not be necessary to use portable air quality sensors to estimate personal exposure. This potential simplification could streamline both data collection and analysis processes, given the inherent complexities associated with deploying portable sensors, both from a participant engagement and data analysis perspective. For those cases, the methodology used in our pilot study, combined with multiple sensors placed in other indoor environments used by participants (e.g., work office), may be sufficient. Overall, our results underscore the importance of both indoor measurements and activity adjusted IR for accurately calculating personal exposure.

On the whole, even though our study was limited by the sample size (seven participants), it demonstrates the significant value of AQSSs in acquiring indoor data for exposure assessment. The widespread use of (calibrated) AQSSs will potentially help to reduce the bias in evaluating gender differences in mortality due to air pollution as indoor measurements reflect more accurately the quality of air to which women and girls are frequently exposed [57]. Furthermore, the granular data collected by AQSSs could drive targeted interventions, enabling policymakers to address specific sources of indoor air pollution, which is not regulated currently, thereby enhancing public health protection and potentially reducing long-term healthcare costs.

## 5. Conclusions

In this work, a pilot project to study the feasibility of using stationary air quality sensors for PM_2.5_ and NO_2_ in epidemiological research was conducted. It was found that the calibrated AQSSs to be used indoors and outdoors at fixed locations were able to run for the month of the study with practically no issues in performance. The results of the exposure assessment showed that using either generic IR or outdoor monitoring station data leads to exposure misclassification. In this study, individuals spent an average of 83% of their time at home. That implies that the use of stationary AQSSs is sufficient for tracking the majority of one’s personal exposure. Future studies could scale up the methodology used here and, using multiple stationary AQSSs, overcome the limitations of this study, as we only measured the air quality in the living room.

A detailed analysis of the indoor and outdoor data measured by the AQSSs in the home of patient 1 revealed a leakage through the window sealing, demonstrating that indoor air quality is influenced not only by routines and behaviours but also by ventilation and building characteristics. Additionally, the source apportionment and activity specific I/O ratio results showed that data from stationary AQSSs, when combined with information from a logbook, can accurately identify and attribute concentration peaks to specific indoor activities. This approach also allows for the evaluation of the influence of outdoor air and ventilation patterns on indoor air quality.

The activity specific concentration and potential dose were calculated and compared with the results of the AIRLESS study. It was shown that there are significant differences in the weight an indoor activity has on personal exposure depending on its intensity. The calculation of the IR, taking into account the information of the activity log, reduces the uncertainty compared with the use of a generic IR in the home environment. 

The results of this study showed consistent indications of symptom intensity fluctuations from day to day, making changes in health status or symptom burden easy to understand and analyse using point scores. Despite varying disease severity, sufficient intra-individual fluctuations were documented to relate day-dependent deviations in symptom burden to air quality data. The examination methods used (health survey, peak flow meter) are therefore considered to be valid for studying the effects of air pollution on vulnerable patient groups with chronic respiratory diseases or asthma. To exclude other influences or confounding variables, a parallel recording of activities and events in the logbook was essential. Overall, this study emphasizes the importance of measuring air quality indoors and tracking activity data for studying personal exposure and how air quality sensors may potentially find good use in the field of environmental epidemiology. 

## Figures and Tables

**Figure 10:**
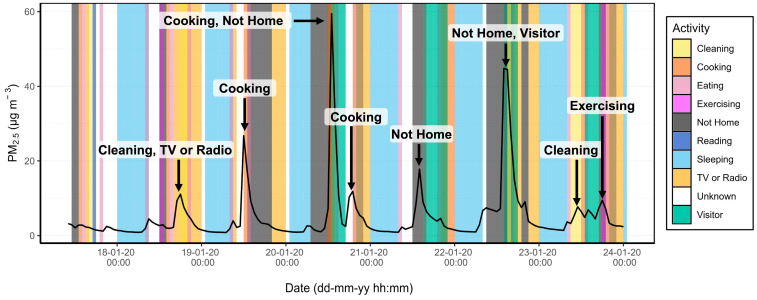
Hourly indoor PM_2.5_ concentration for patient 2 from 18 January to 24 January 2020, combined with recorded activities.

**Table 1:** Groups of activities tracked by patients and assigned default values.

Category	Possible Values	Default Value
Patient location	Home Not home Garden or balcony	Home
Window status	Window closed Window tilted Window open	Window closed
Activity	Sleeping Exercising Reading Computer TV or radio Cooking Eating Visitor Cleaning	Unknown

**Table 2:** Mean, minimum, and maximum health score and PEF values for all participants.

Patient ID	Health Score (0–28)			PEF (L min^−1^)		
	Minimum	Mean	Maximum	Minimum	Mean	Maximum
1	10	14.5	20	-	-	-
2	-	-	-	-	-	-
3	6	11.1	18	500	540	580
4	0	1.6	4	370	400	430
5	-	-	-	-	-	-
6	0	1.0	5	800	800	800
7	0	1.2	4	290	324	370

**Table 3:** Average of the generic and activity adjusted hourly IR for individual patients (excluding sleeping hours).

Patient ID	Hourly Mean IR (L min^−1^)	
	Activity Adjusted	Generic
1	7.0	9
2	9.2	6.8
3	8.3	9.9
4	5.3	8.5
6	9.5	12.1
7	9.5	8.5

## Data Availability

The raw data supporting the conclusions of this article will be made available by the authors upon request.

## Supplementary Materials

The following supporting information can be downloaded at: https://www.mdpi.com/article/10.3390/s24175767/s1, Table S1: Dates of AQSS installation, checks, and collection for each patient; Table S2: Participant demographics; Figure S1: (a) Map of Stuttgart showing locations of the outdoor air quality monitoring station (blue circle) and participants’ homes where sensors were deployed (yellow diamonds), (b) outdoor AQSS and (c) indoor AQSS; Table S3: Data collected for each participant; Table S4: Selected results of the environmental questionnaire filled out by patients; Figure S2: Logbook; Table S5: Data completeness after cleaning steps, presented as a percentage; Table S6: Correction parameters for each PM_2.5_ sensor; Figure S3: Health questionnaire; Table S7: Inhalation rates for each intensity level, categorised by associated activities, age and gender; Table S8: Median NO_2_ and PM_2.5_ concentrations and uncertainties associated with each AQSS; Figure S4: I/O ratios for PM_2.5_ associated with individual activities, grouped by window status for patient 7; Figure S5: Time series of pollutant concentration combined with logged activities and window status, for patient 1, week 1; Figure S6: Time series of pollutant concentration combined with logged activities and window status, for patient 1, week 2; Figure S7: Time series of pollutant concentration combined with logged activities and window status, for patient 1, week 3; Figure S8: Time series of pollutant concentration combined with logged activities and window status, for patient 1, week 4; Figure S9: Time series of pollutant concentration combined with logged activities and window status, for patient 2, week 1; Figure S10: Time series of pollutant concentration combined with logged activities and window status, for patient 2, week 3; Figure S11: Time series of pollutant concentration combined with logged activities and window status, for patient 2, week 4; Figure S12: Time series of pollutant concentration combined with logged activities and window status, for patient 3, week 1; Figure S13: Time series of pollutant concentration combined with logged activities and window status, for patient 3, week 2; Figure S14: Time series of pollutant concentration combined with logged activities and window status, for patient 3, week 3; Figure S15: Time series of pollutant concentration combined with logged activities and window status, for patient 3, week 4; Figure S16: Time series of pollutant concentration combined with logged activities and window status, for patient 4, week 1; Figure S17: Time series of pollutant concentration combined with logged activities and window status, for patient 4, week 2; Figure S18: Time series of pollutant concentration combined with logged activities and window status, for patient 4, week 3; Figure S19: Time series of pollutant concentration combined with logged activities and window status, for patient 6, week 1; Figure S20: Time series of pollutant concentration combined with logged activities and window status, for patient 6, week 2; Figure S21: Time series of pollutant concentration combined with logged activities and window status, for patient 6, week 3: Note that the outdoor sensor stopped working on 19 April 2020 at 3:00 am; Figure S22: Time series of pollutant concentration combined with logged activities and window status, for patient 6, week 4; Figure S23: Time series of pollutant concentration combined with logged activities and window status, for patient 7, week 1; Figure S24: Time series of pollutant concentration combined with logged activities and window status, for patient 7, week 2; Figure S25: Time series of pollutant concentration combined with logged activities and window status, for patient 7, week 3; Figure S26: Time series of pollutant concentration combined with logged activities and window status, for patient 7, week 4; Figure S27: Daily indoor and outdoor PM_2.5_ concentrations during PM alert (from 21 January to 26 January 2020) during the measurement campaign in the home of patient 1; Figure S28: Individual activity specific PM_2.5_ (left) and NO_2_ (right) concentrations, for patients 6 and 7: Note that the PM_2.5_ concentrations are on a logarithmic scale; Figure S29: Individual activity specific PM_2.5_ (left) and NO_2_ (right) concentrations, for patients 1, 2, and 3 and 4: Note that the PM_2.5_ concentrations are on a logarithmic scale; Figure S30: Hourly indoor and outdoor (garden and street) PM_2.5_ and NO_2_ concentrations, self-reported health score and PEF for patient 4; Figure S31: Hourly indoor and outdoor PM_2.5_ and NO_2_ concentrations, self-reported health score and PEF for patient 6: Note that the outdoor sensor stopped working on 19 April 2020 at 3:00 am; Figure S32: Hourly indoor and outdoor PM_2.5_ and NO_2_ concentrations, self-reported health score and PEF for patient 7.
