# Supplementary material for: Feasibility Study on the Use of NO2 and PM2.5 Sensors for Exposure Assessment and Indoor Source Apportionment at Fixed Locations
Source: Sensors (Basel). 2024 Sep 5;24(17):5767. doi: 10.3390/s24175767 (PMC11398243; doi:10.3390/s24175767)
Supplement: Supplementary file 1 [file sensors-24-05767-s001.zip › sensors-3137902-supplementary.pdf]

Supplementary material

Feasibility study on the use of NO_2 and $PM_{2.5}$ sensors for exposure assessment and indoor source apportionment at fixed locations

Miriam Chacón-Mateos[1*], Erika Remy[2], Uta Liebers[3,4], Frank Heimann[5], Christian Witt[3], Ulrich Vogt[1]

[1]University of Stuttgart, Department of Flue Gas Cleaning and Air Quality Control, 70569 Stuttgart, Germany
[2]University of Hohenheim, Institute of Physics and Meteorology, 70599 Stuttgart, Germany
[3]Charité - Universitätsmedizin Berlin, Institute of Physiology, Corporate Member of Freie Universität Berlin and Humboldt-Universität zu Berlin, 10117 Berlin, Germany
[4]Department of Pneumology, Evangelische Lungenklinik Berlin Buch, 13125 Berlin, Germany
[5]Ambulante Pneumologie mit Allergie Zentrum, 70178 Stuttgart, Germany

Correspondence to: Miriam Chacón-Mateos (Miriam.Chacon-Mateos@ifk.uni-stuttgart.de)

Table S1. Dates of AQSS installation, checks, and collection for each patient.

		Patient ID						
		1	2	3	4	5	6	7
Indoor	Start	Jan. 8, 2020	Jan. 17, 2020	Mar. 9, 2020	Mar. 9, 2020	Dec. 20, 2019	Apr. 3, 2020	Apr. 29, 2020
	Mid-way Check	-	-	Mar. 24, 2020	-	-	Apr. 11, 2020	May 12, 2020
	End	Feb. 6, 2020	Feb. 14, 2020	Apr. 7, 2020	Mar. 27, 2020	Jan. 15, 2020	Apr. 30, 2020	May 28, 2020
	Total days	30	29	30	19	27[a)]	28	30
Outdoor	Start	Jan. 13, 2020	Jan. 24, 2020	Mar. 9, 2020	Mar. 13, 2020	-	Apr. 3, 2020	Apr. 29, 2020
	Mid-way Check	-	-	Mar. 24, 2020	-	-	Apr. 11, 2020	May 12, 2020
	End	Feb. 6, 2020	Feb. 14, 2020	Apr. 7, 2020	Mar. 27, 2020	-	Apr. 30, 2020	May 28, 2020
	Total days	25	22[b)]	30	15[c)]	-	28	30

[a)] Patient disconnected the AQSS from the power so that only 9 days were recorded.
[b)] All data was lost due to a storm.
[c)] Two AQSS were installed, one in the garden and the other at the street side.

Table S2. Participant demographics.

Patient ID	Age Group	Sex	Diagnosis
1	71 - 80	M	COPD II/B
2	71 - 80	F	Asthma
3	61 - 70	M	COPD II/B
4	41 - 50	F	Asthma
5	41 - 50	F	Asthma
6	21 - 30	M	Asthma
7	51 - 60	F	Asthma

M: Male, F: Female.

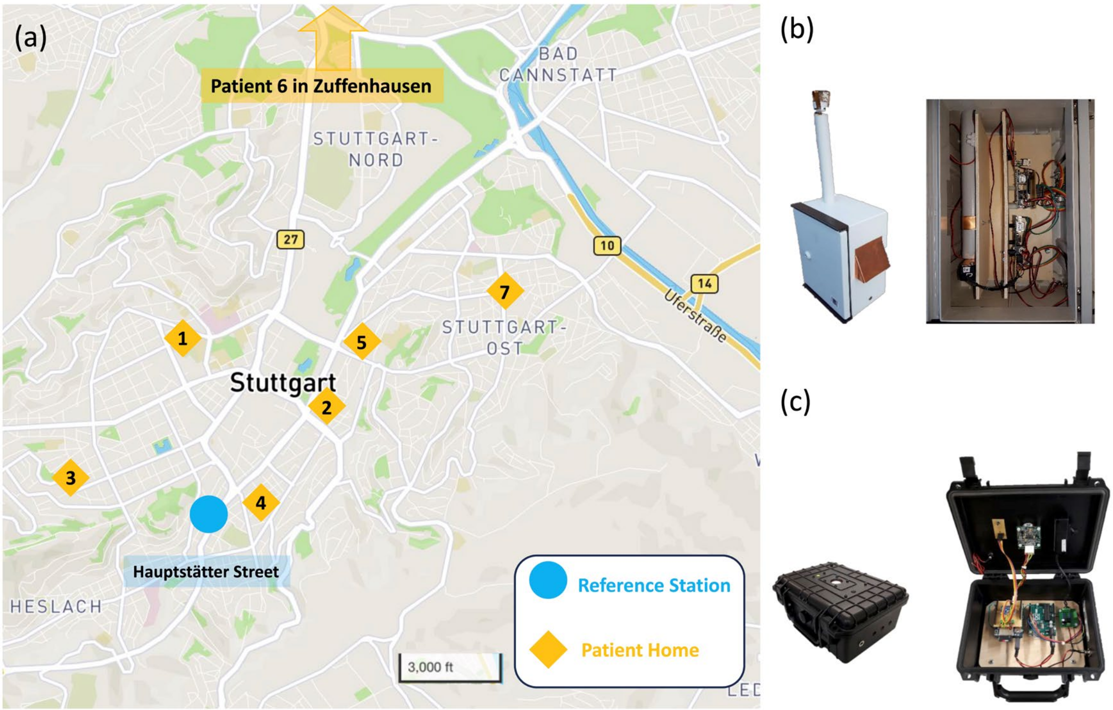

Map source: AllTrails

Figure S1. (a) Map of Stuttgart showing locations of the outdoor air quality monitoring station (blue circle) and participants' homes where sensors were deployed (yellow diamonds), (b) outdoor AQSS and (c) indoor AQSS.

Table S3. Data collected for each participant.

Pat. ID	Type of Data					
	Indoor AQSS	Outdoor AQSS	Logbook	Health Questionnaire	Environmental Survey	Feedback
1	✓	✓	✓	✓	✓	✓
2	✓	X [a)]	✓	X [b)]	✓	✓
3	✓	✓	✓	✓	✓	✓
4	✓	✓ [c)]	✓	✓	✓	✓
5	✓	X [d)]	X [b)]	X [b)]	✓	✓
6	✓	✓	✓	✓	✓	✓
7	✓	✓	✓	✓	✓	✓

[a)] Outdoor sensor data lost due to storm.
[b)] Insufficient data recorded by the participant.
[c)] Two outdoor sensor systems on opposite sides of the house (street and garden).
[d)] No outdoor sensor was installed.

Table S4. Selected results of the environmental questionnaire filled out by patients.

		Patient ID						
		1	2	3	4	5	6	7
Surrounding Area	Landscape Surrounding House	in a valley	in a valley	on a hillside	on a hillside	in a valley	in a depression	in a depression
	Type of Street (side/main)	side	side	main	side	side	side	side
	Distance to Busy Road (m)	870	80	16	433	60	100 - 500	> 500
	Construction Near	Yes	Yes	No	No	Yes	No	No
Apartment Layout	Floor Level	2	3	1	Basement	Ground floor and 1	Ground floor	1
	Garden or Balcony	No	Yes	Yes Balcony	Yes Balcony	Yes Garden	Yes Garden	Yes Garden and Balcony
	Apartment Area (m^2)	50	53	90	60	87	75	89
	Area of Room where Sensor is Placed (m^2)	28	36	36	55	15	16	15-20
	Open Kitchen	No	Yes	No	No	No	No	No
Utilities	Type of Stove	Electric	Electric	Gas	Electric	Electric	Electric	Electric
	Air Conditioning	No	No	No	No	Yes	No	No
	Type of Heating	District Heating	Oil	Gas - Single Boilers	District Heating	Gas - Central Heating	Central Heating	Central Heating
	Number of Additional Residents	0	0	1	0	3	1	1

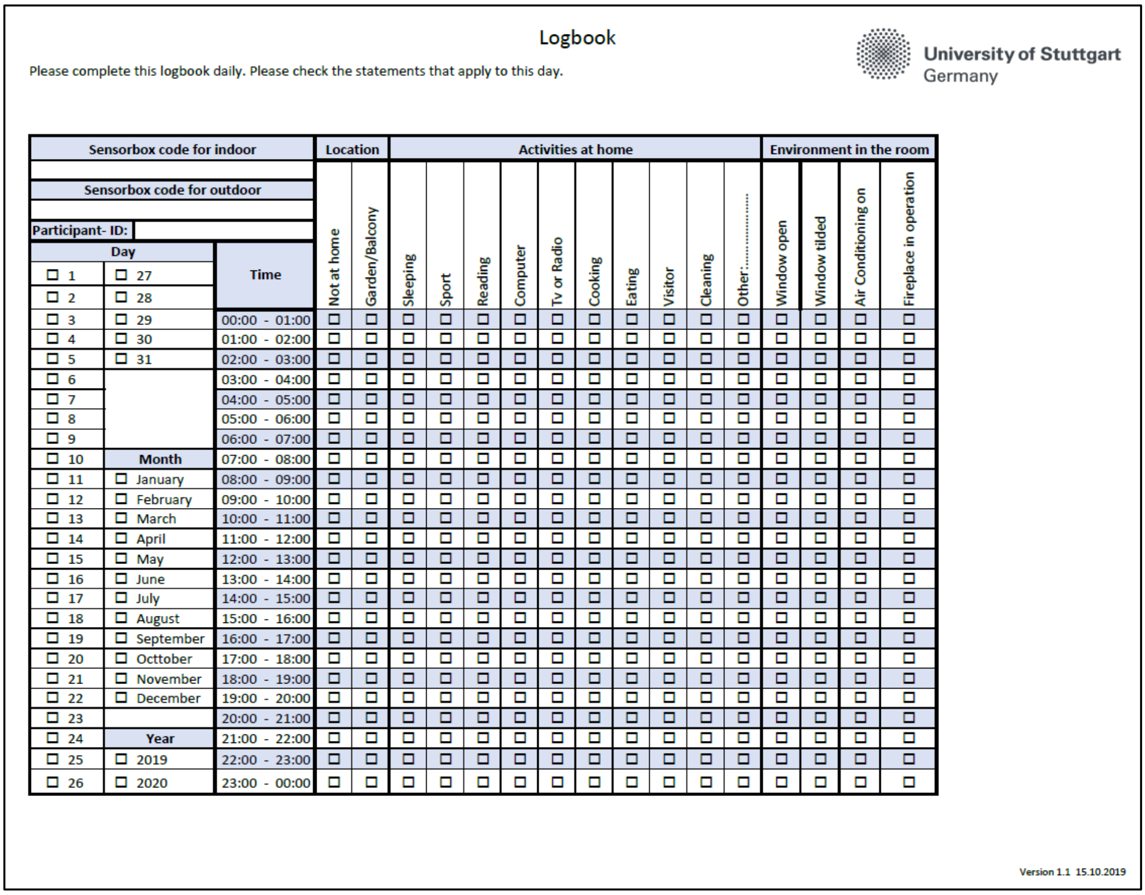

Logbook

University of Stuttgart Germany

Please complete this logbook daily. Please check the statements that apply to this day.

Sensorbox code for indoor

Sensorbox code for outdoor

Participant- ID:

			Location		Activities at home										Environment in the room			
Day		Time	Not at home	Garden/Balcony	Sleeping	Sport	Reading	Computer	Tv or Radio	Cooking	Eating	Visitor	Cleaning	Other:.........	Window open	Window tilded	Air Conditioning on	Fireplace in operation
☐ 1	☐ 27																	
☐ 2	☐ 28																	
☐ 3	☐ 29	00:00 - 01:00	☐	☐	☐	☐	☐	☐	☐	☐	☐	☐	☐	☐	☐	☐	☐	☐
☐ 4	☐ 30	01:00 - 02:00	☐	☐	☐	☐	☐	☐	☐	☐	☐	☐	☐	☐	☐	☐	☐	☐
☐ 5	☐ 31	02:00 - 03:00	☐	☐	☐	☐	☐	☐	☐	☐	☐	☐	☐	☐	☐	☐	☐	☐
☐ 6		03:00 - 04:00	☐	☐	☐	☐	☐	☐	☐	☐	☐	☐	☐	☐	☐	☐	☐	☐
☐ 7		04:00 - 05:00	☐	☐	☐	☐	☐	☐	☐	☐	☐	☐	☐	☐	☐	☐	☐	☐
☐ 8		05:00 - 06:00	☐	☐	☐	☐	☐	☐	☐	☐	☐	☐	☐	☐	☐	☐	☐	☐
☐ 9		06:00 - 07:00	☐	☐	☐	☐	☐	☐	☐	☐	☐	☐	☐	☐	☐	☐	☐	☐
☐ 10	Month	07:00 - 08:00	☐	☐	☐	☐	☐	☐	☐	☐	☐	☐	☐	☐	☐	☐	☐	☐
☐ 11	☐ January	08:00 - 09:00	☐	☐	☐	☐	☐	☐	☐	☐	☐	☐	☐	☐	☐	☐	☐	☐
☐ 12	☐ February	09:00 - 10:00	☐	☐	☐	☐	☐	☐	☐	☐	☐	☐	☐	☐	☐	☐	☐	☐
☐ 13	☐ March	10:00 - 11:00	☐	☐	☐	☐	☐	☐	☐	☐	☐	☐	☐	☐	☐	☐	☐	☐
☐ 14	☐ April	11:00 - 12:00	☐	☐	☐	☐	☐	☐	☐	☐	☐	☐	☐	☐	☐	☐	☐	☐
☐ 15	☐ May	12:00 - 13:00	☐	☐	☐	☐	☐	☐	☐	☐	☐	☐	☐	☐	☐	☐	☐	☐
☐ 16	☐ June	13:00 - 14:00	☐	☐	☐	☐	☐	☐	☐	☐	☐	☐	☐	☐	☐	☐	☐	☐
☐ 17	☐ July	14:00 - 15:00	☐	☐	☐	☐	☐	☐	☐	☐	☐	☐	☐	☐	☐	☐	☐	☐
☐ 18	☐ August	15:00 - 16:00	☐	☐	☐	☐	☐	☐	☐	☐	☐	☐	☐	☐	☐	☐	☐	☐
☐ 19	☐ September	16:00 - 17:00	☐	☐	☐	☐	☐	☐	☐	☐	☐	☐	☐	☐	☐	☐	☐	☐
☐ 20	☐ Octtober	17:00 - 18:00	☐	☐	☐	☐	☐	☐	☐	☐	☐	☐	☐	☐	☐	☐	☐	☐
☐ 21	☐ November	18:00 - 19:00	☐	☐	☐	☐	☐	☐	☐	☐	☐	☐	☐	☐	☐	☐	☐	☐
☐ 22	☐ December	19:00 - 20:00	☐	☐	☐	☐	☐	☐	☐	☐	☐	☐	☐	☐	☐	☐	☐	☐
☐ 23		20:00 - 21:00	☐	☐	☐	☐	☐	☐	☐	☐	☐	☐	☐	☐	☐	☐	☐	☐
☐ 24	Year	21:00 - 22:00	☐	☐	☐	☐	☐	☐	☐	☐	☐	☐	☐	☐	☐	☐	☐	☐
☐ 25	☐ 2019	22:00 - 23:00	☐	☐	☐	☐	☐	☐	☐	☐	☐	☐	☐	☐	☐	☐	☐	☐
☐ 26	☐ 2020	23:00 - 00:00	☐	☐	☐	☐	☐	☐	☐	☐	☐	☐	☐	☐	☐	☐	☐	☐

Version 1.1 15.10.2019

Figure S2. Logbook.

Table S5. Data completeness after cleaning steps, presented as a percentage.

	$PM_{2.5}$		NO_2		Symptom severity	
**Patient ID**	**Indoor**	**Outdoor**	**Indoor**	**Outdoor**	**Health Score**	**PEF**
1	100	80	97	94	100	0
2	100	-	99	-	0	0
3	99	99	86	91	99	99
4	100	100 / 100[a)]	78	94 / 83 [a)]	94	94
5	92	-	82	-	0	0
6	100	100	94	92	91	91
7	99	100	93	76	99	92

[a)] For patient 4 two outdoor AQSS are reported: Garden / Street.

Table S6. Correction parameters for each $PM_{2.5}$ sensor.

Location	Patient ID	AQSS ID	Correction Factor, *m*	Offset, *b* (μg m^{-3})
INDOOR	1	B02	1.15	-0.14
	2	B04	0.89	0.42
	3	B04	2.34	-1.38
	5	B01	0.97	0.20
	6	B02	1.01	3.24
	7	B03	0.93	2.33
	4	B01	1.06	1.74
OUTDOOR	1	B03	0.83	4.66
	2[a)]	B05	0.37	8.96
	3	B03	0.93	2.33
	5 [b)]	-	-	-
	6	B08	0.86	1.40
	7	B06	0.79	2.92
	4 (Garden)	B05	0.96	1.04
	4 (Street)	B06	0.55	1.15

[a)] An outdoor AQSS was deployed for patient 2, but data was lost due to a storm.

[b)] No outdoor sensor was installed.

CHARITÉ
UNIVERSITÄTSMEDIZIN BERLIN

Patient Diary

Dear patient,

Please complete this short questionnaire every evening. Check the statements that apply to this day.

DATE……………………………………………………………**Patient-ID**……………………………………

Parameter	Please mark with a cross where applicable	Points
Did you feel a tightness in your chest today?	0 none not at all<br>1 mild only once and for a short time<br>2 moderate multiple short-term<br>3 strong several times longer lasting<br>4 very strong lasting for hours	
How difficult was it for you to breathe today??	0 none no breathing difficulties at all<br>1 mild shortness of breath only with exceptional exertion (e.g. sport)<br>2 moderate short of breath with little exertion (short flight of stairs)<br>3 Strong short of breath even with light physical activity (walking on level ground)<br>4 very strong constantly short of breath, even at rest	
How was your cough today?	0 none not coughed<br>1 hardly coughed once briefly<br>2 occasionally coughed briefly several times<br>3 frequently coughing almost every hour<br>4 constantly coughed all the time or felt the urge to cough	
How annoying was your sputum today?	0 no mucus formation at all<br>1 hardly any mucus, coughed up once<br>2 Slightly rarely coughed up, mucus perceived as annoying<br>3 very frequent, unpleasant mucus production<br>4 very strong bothered me the whole day	
Have you noticed a whistling breathing noise?	0 none not noticed any breathing noises<br>1 hardly once and for a short time<br>2 occasionally several times a day<br>3 frequently several times and over a longer period<br>4 constantly for most of the day	
Has your lung disease prevented you from doing as much as usual at work, at university or at home?	0 none no impairment at all<br>1 hardly only particularly strenuous activities (e.g. sport)<br>2 occasionally some activities required a little more time<br>3 frequently most activities were carried out more slowly, strenuous activities were postponed<br>4 constantly very limited, only the bare essentials done	
How often have you used your on-demand spray today?	0 none not needed<br>1 hardly needed once a day<br>2 rarely needed 2 times a day<br>3 often needed 3 times a day<br>4 very often needed more than 3 times a day	
Please enter your evening peak flow value (before the evening dose of your spray or powder)	------------------------	**Total**

Version 1.3. 21.08.2019

Figure S3. Health questionnaire.

Table S7. Inhalation rates for each intensity level, categorised by associated activities, age and gender.

Intensity Level	Activities	Age Range (Years)	Inhalation Rate (L min^{-1}) Male	Female
RESTING	Sleeping	20-30	4.7	3.9
		40-50	5.7	4.4
		50-60	5.8	4.6
		60-70	6.0	4.5
		70-80	6.1	4.5
SEDENTARY	Reading<br>Computer<br>Television<br>Radio<br>Eating<br>Smoking	20-30	5.1	4.3
		40-50	6.1	4.8
		50-60	6.3	5.0
		60-70	6.5	4.9
		70-80	6.7	5.0
LIGHT	Cooking<br>Cleaning	20-30	13.0	10.6
		40-50	14.4	11.8
		50-60	14.6	12.0
		60-70	14.1	10.8
		70-80	13.9	10.8
MODERATE	Sport /Exercising	20-30	29.2	22.9
		40-50	31.6	24.5
		50-60	32.7	25.2
		60-70	29.8	21.4
		70-80	29.3	21.1
GENERIC	Unknown	20-30	12.1	9.3
		40-50	11.3	8.5
		50-60	11.3	8.5
		60-70	9.9	7.8
		70-80	9.0	6.8

Note: Activity adjusted inhalation rates are provided in the Exposure Factors Handbook (2011) [41], Table 6-17 (for males), and 6-19 (for females). Generic inhalation rates for males and females are provided in Table 6-5.

Table S8. Median NO_2 and $PM_{2.5}$ concentrations and uncertainties associated with each AQSS.

Pollutant	Patient ID	AQSS ID	Location	Median concentration (µg m^{-3})	Relative expanded uncertainty at the median (µg m^{-3})
NO_2	1	B02	Indoor	28	14
	2	B04	Indoor	9	1
	3	B04	Indoor	13	1
	4	B01	Indoor	5	1
	5	B01	Indoor	13	1
	6	B02	Indoor	6	4
	7	B03	Indoor	19	6
	1	B03	Outdoor	23	4
	2	B05	Outdoor	-	-
	3	B03	Outdoor	21	6
	4	B05	Garden	35	13
	4	B06	Street	29	7
	5	-	-	-	-
	6	B08	Outdoor	20	5
	7	B06	Outdoor	14	6
$PM_{2.5}$	1	B02	Indoor	3	12
	2	B04	Indoor	2	8
	3	B04	Indoor	5	1
	4	B01	Indoor	4	11
	5	B01	Indoor	2	12
	6	B02	Indoor	6	11
	7	B03	Indoor	57	7
	1	B03	Outdoor	11	7
	2	B05	Outdoor	-	-
	3	B03	Outdoor	11	7
	4	B05	Garden	6	7
	4	B06	Street	7	2
	5	-	-	-	-
	6	B08	Outdoor	5	21
	7	B06	Outdoor	7	2

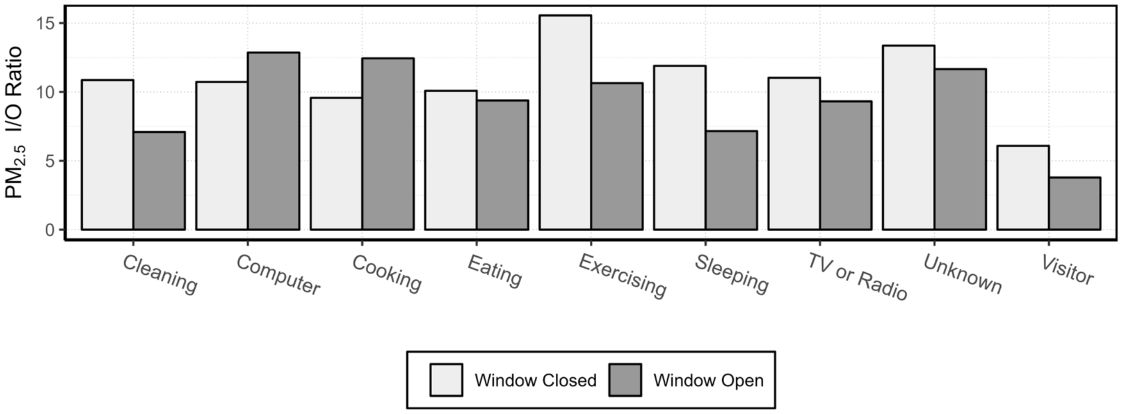

Figure S4. I/O ratios for $PM_{2.5}$ associated with individual activities, grouped by window status for patient 7.

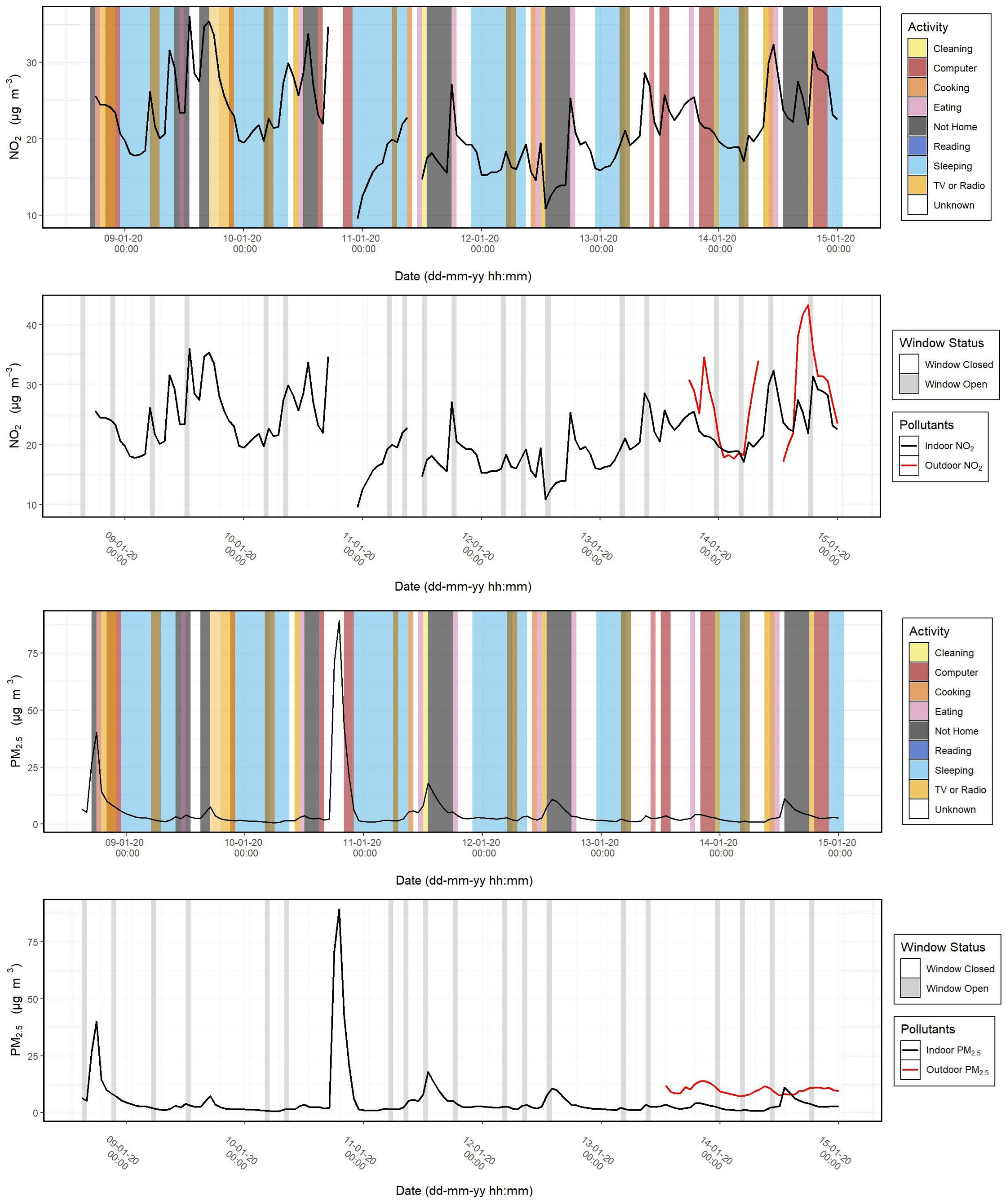

Figure S5. Time series of pollutant concentration combined with logged activities and window status, for patient 1, week 1.

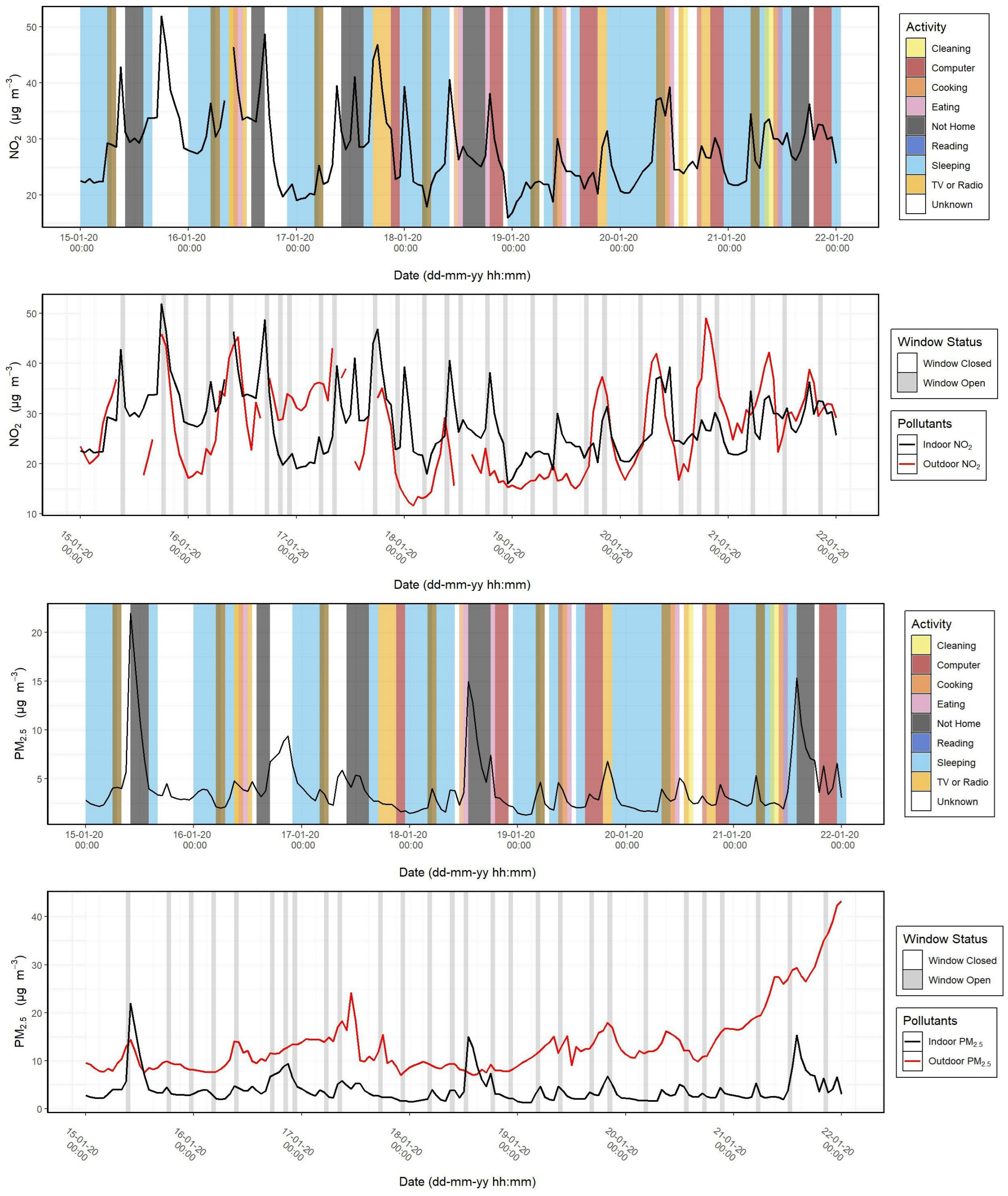

Figure S6. Time series of pollutant concentration combined with logged activities and window status, for patient 1, week 2.

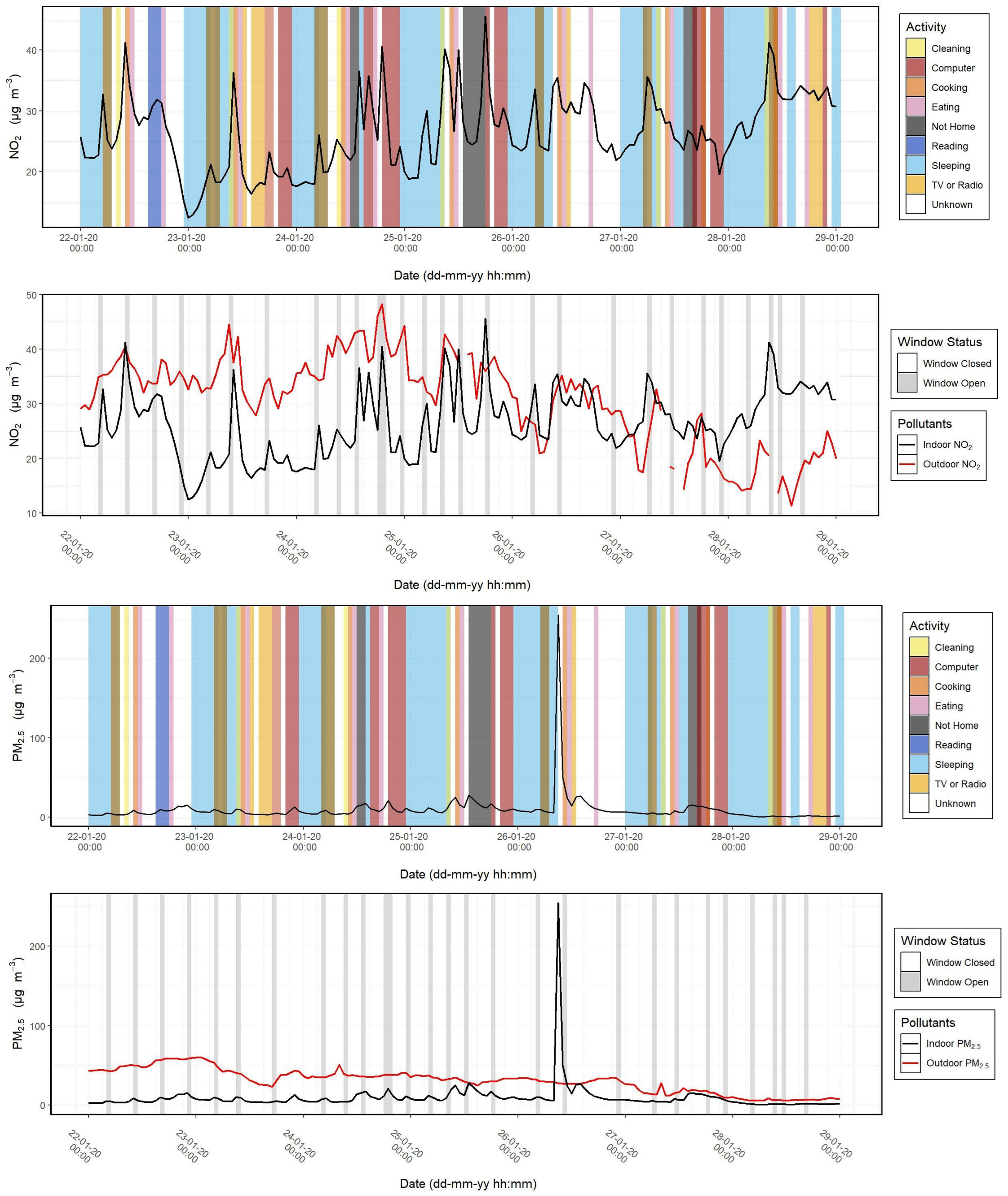

Figure S7. Time series of pollutant concentration combined with logged activities and window status, for patient 1, week 3.

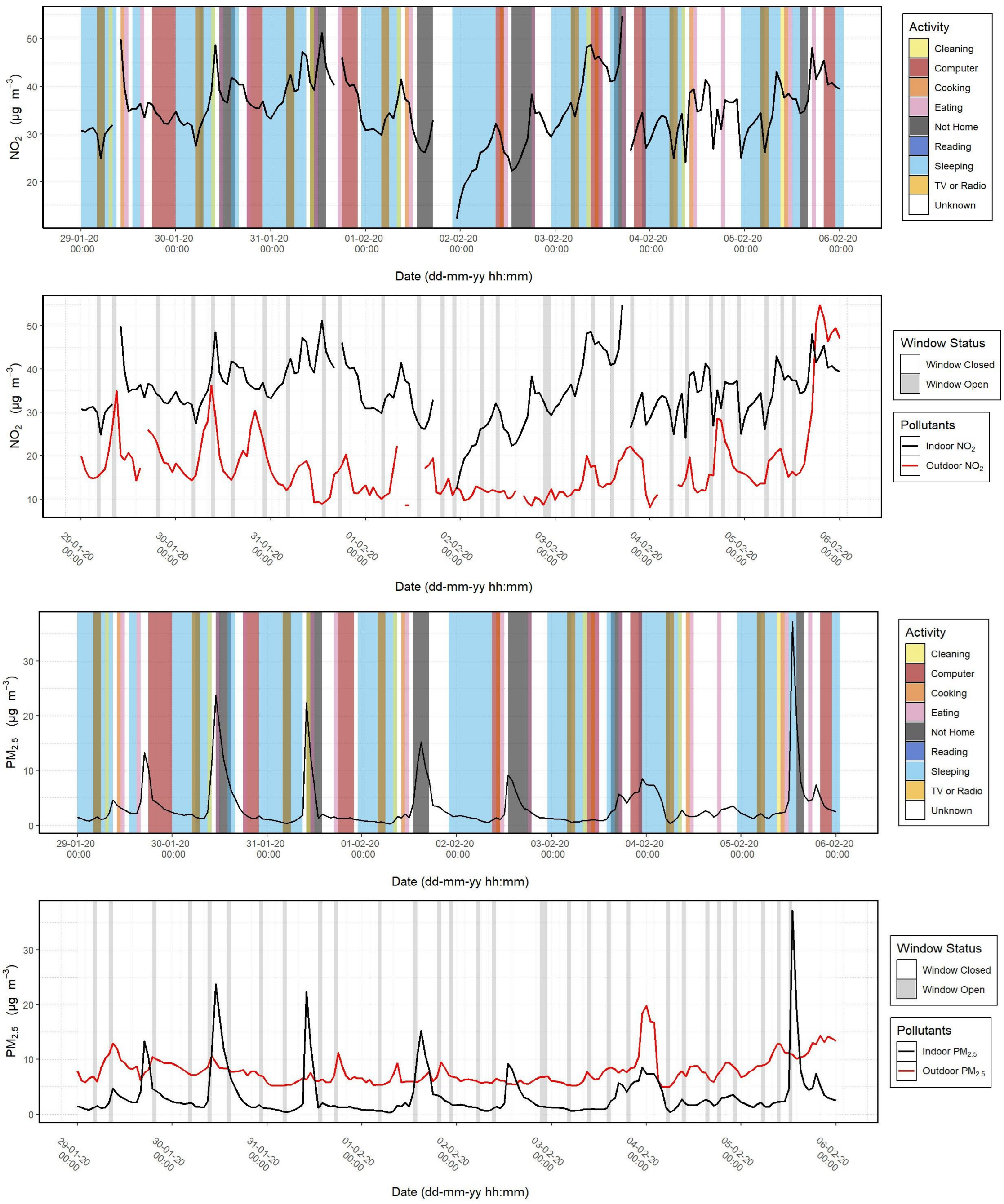

Figure S8. Time series of pollutant concentration combined with logged activities and window status, for patient 1, week 4.

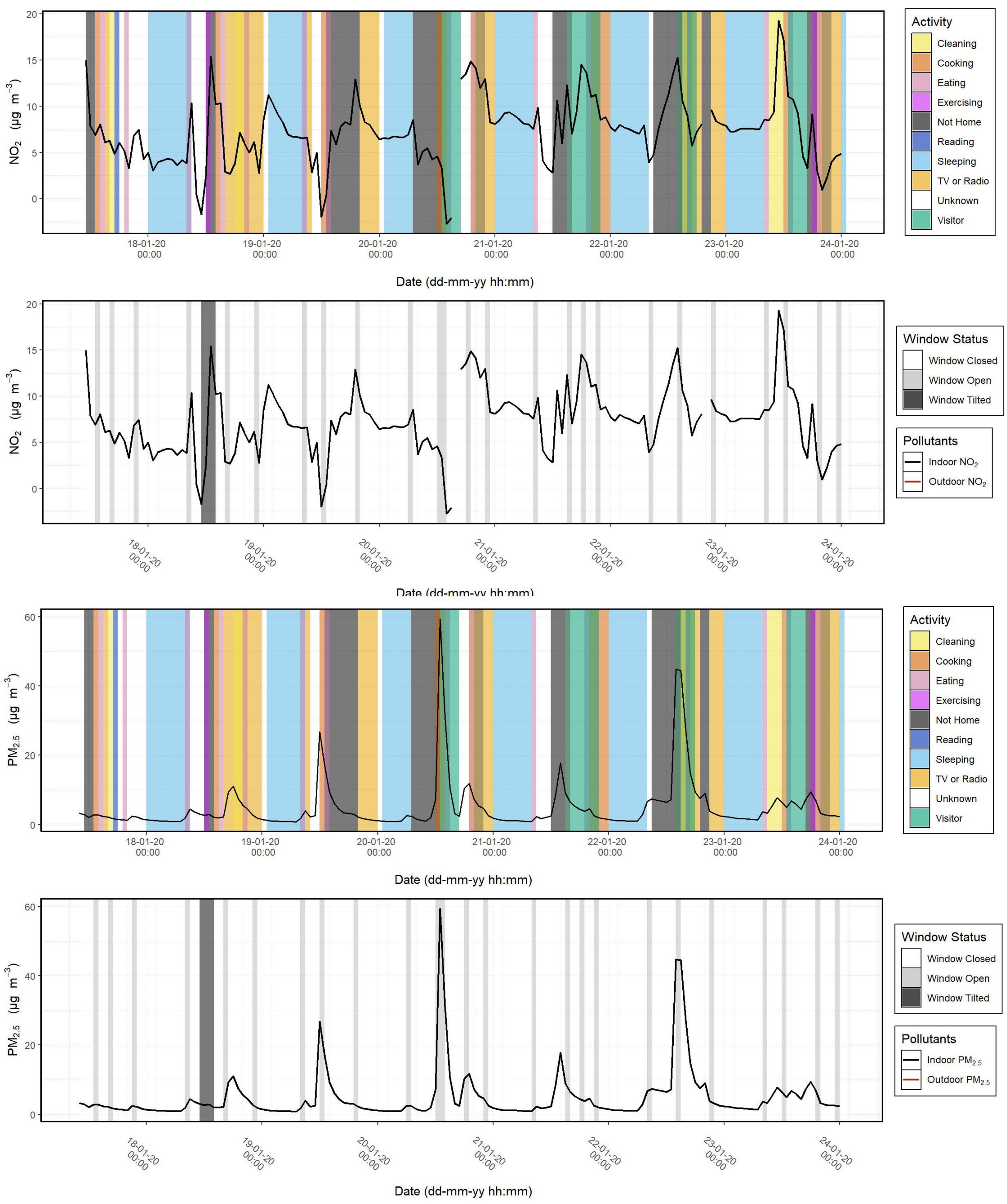

Figure S9. Time series of pollutant concentration combined with logged activities and window status, for patient 2, week 1.

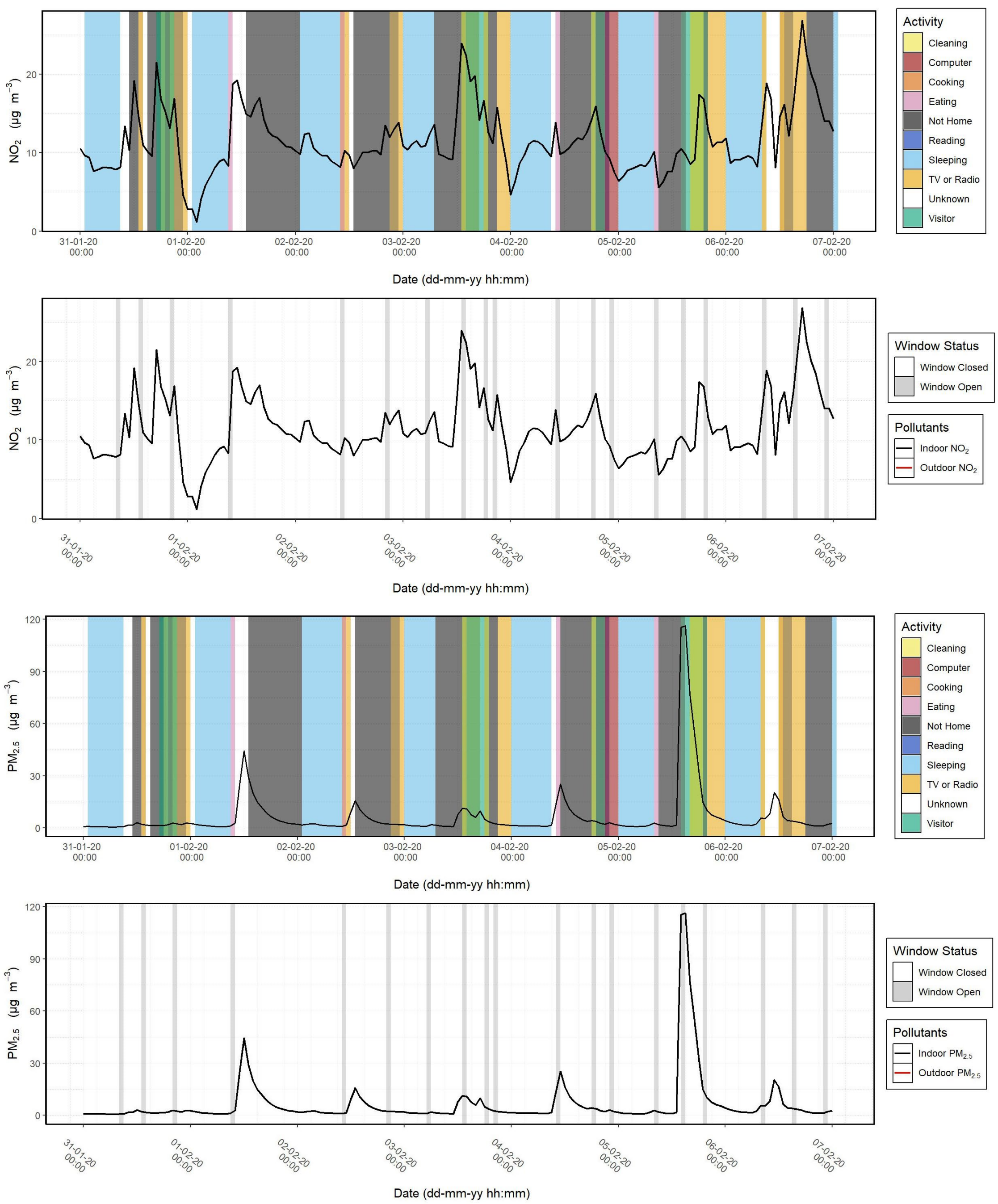

Figure S10. Time series of pollutant concentration combined with logged activities and window status, for patient 2, week 3.

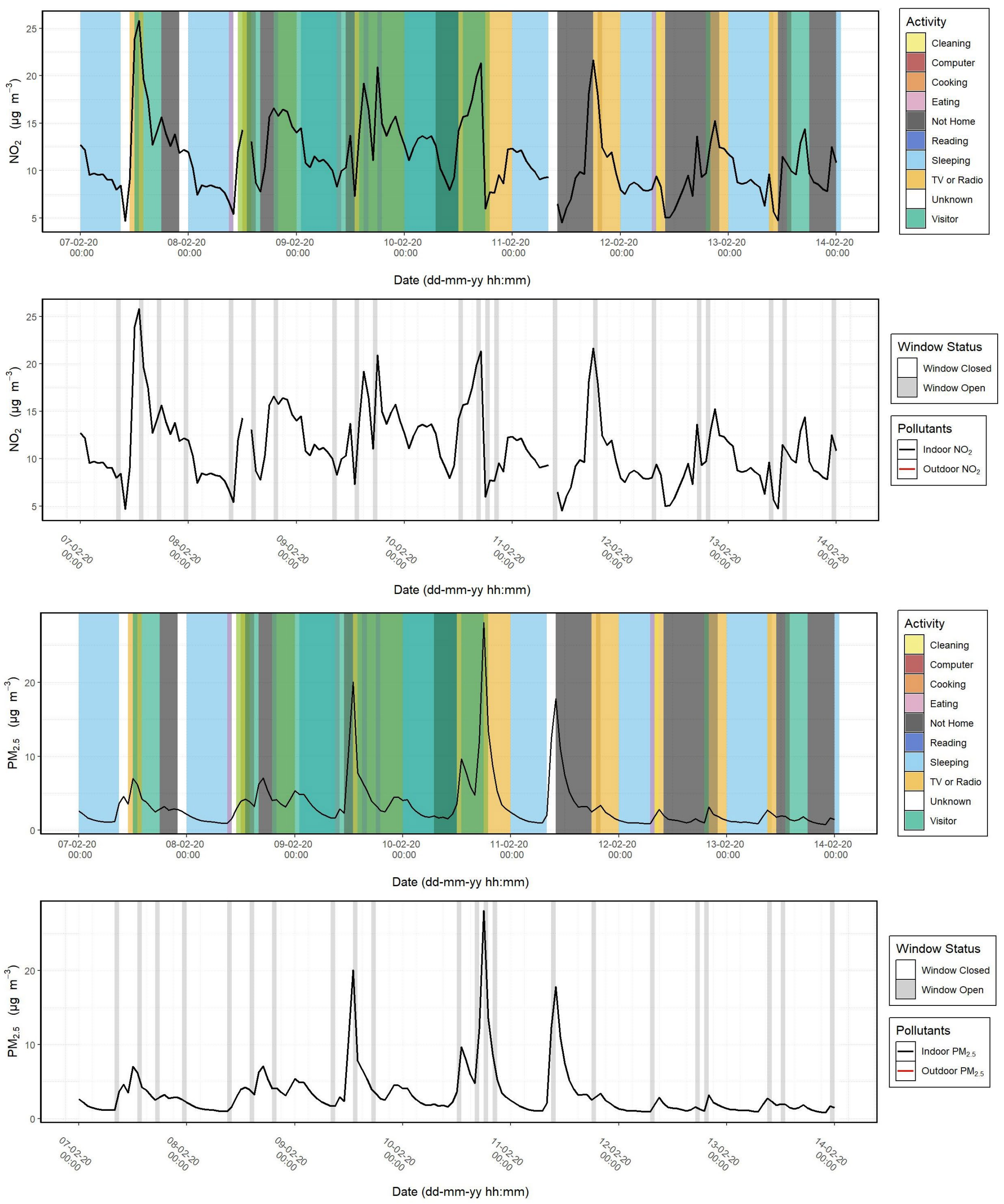

Figure S11. Time series of pollutant concentration combined with logged activities and window status, for patient 2, week 4.

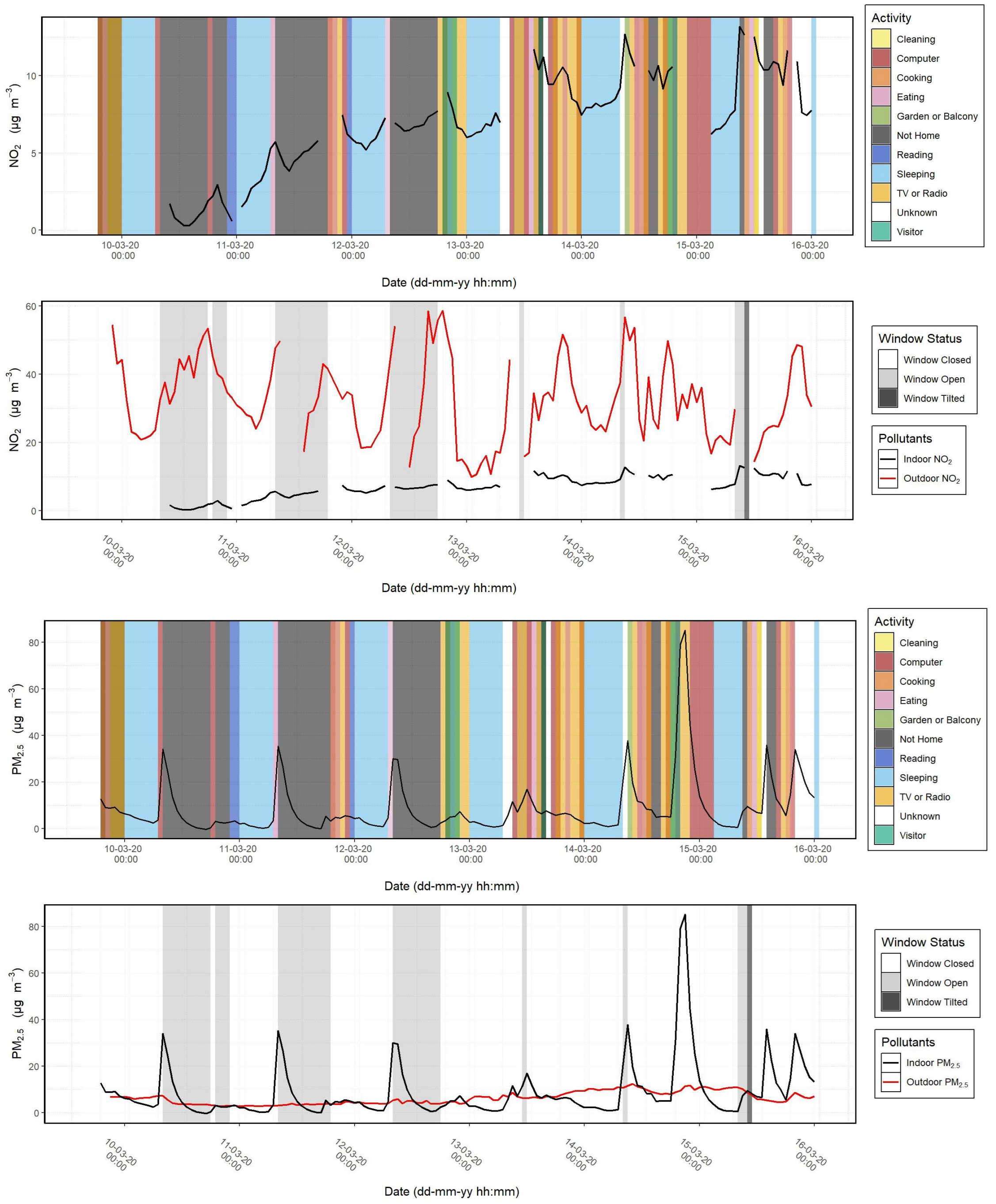

Figure S12. Time series of pollutant concentration combined with logged activities and window status, for patient 3, week 1.

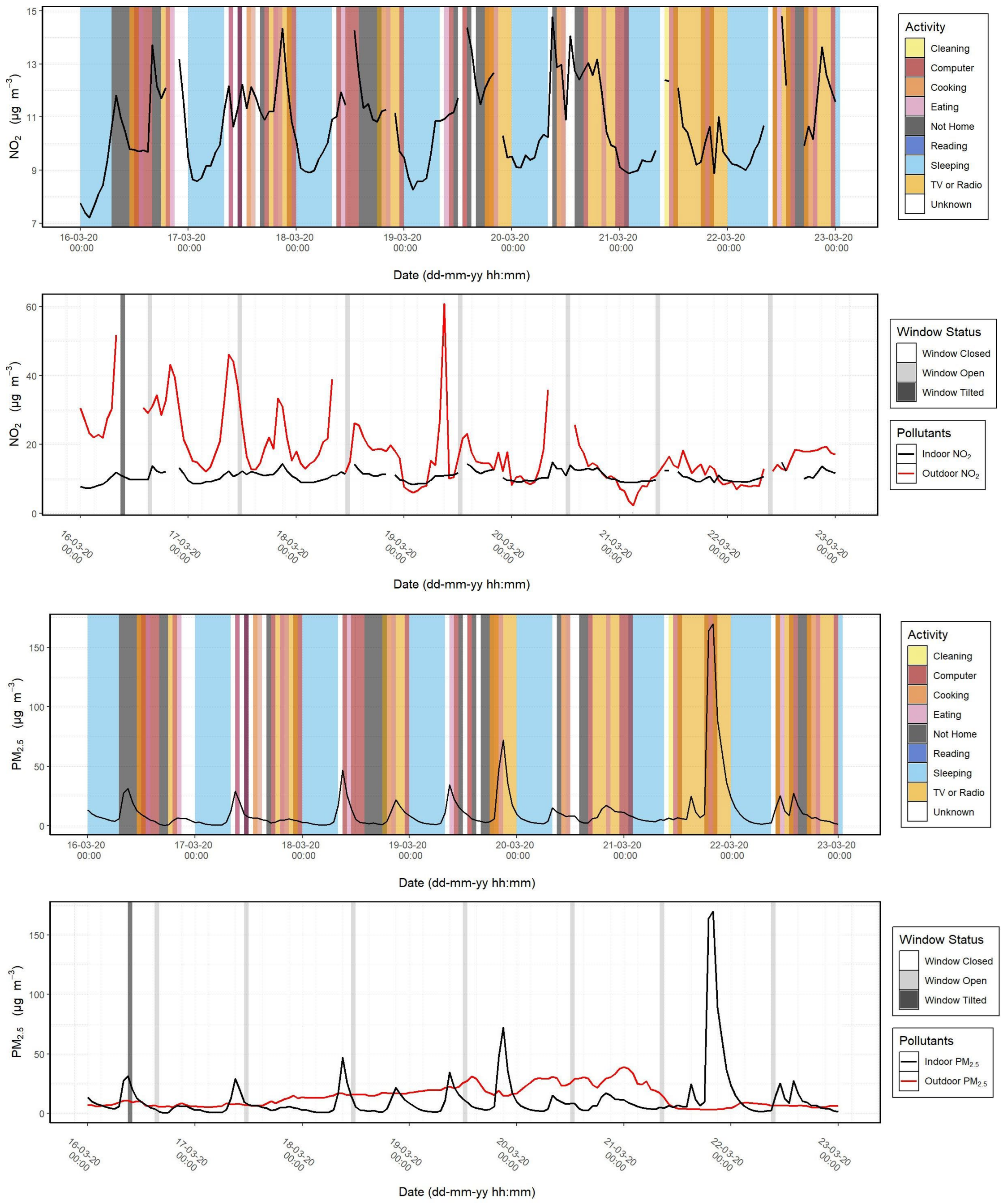

Figure S13. Time series of pollutant concentration combined with logged activities and window status, for patient 3, week 2.

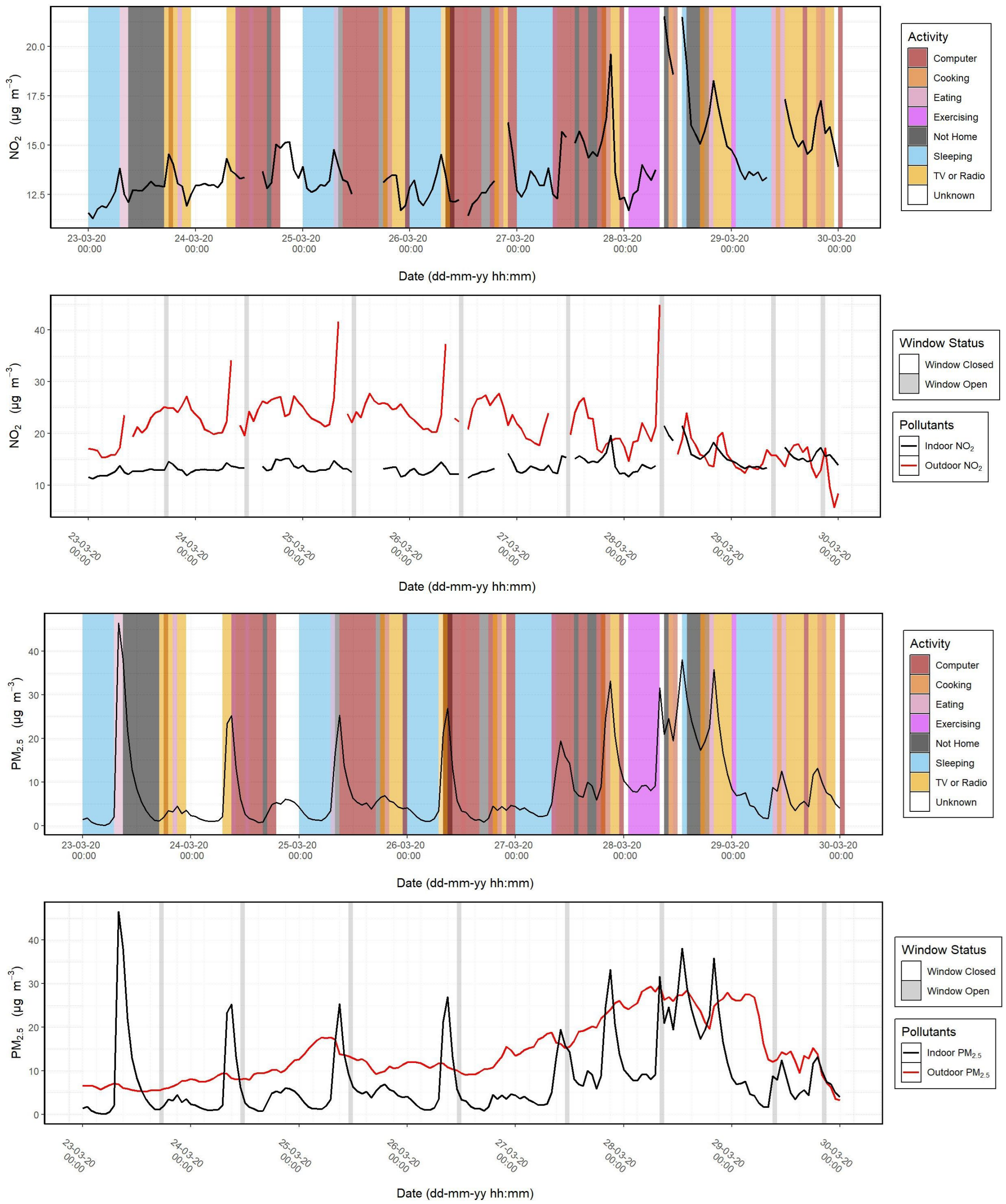

Figure S14. Time series of pollutant concentration combined with logged activities and window status, for patient 3, week 3.

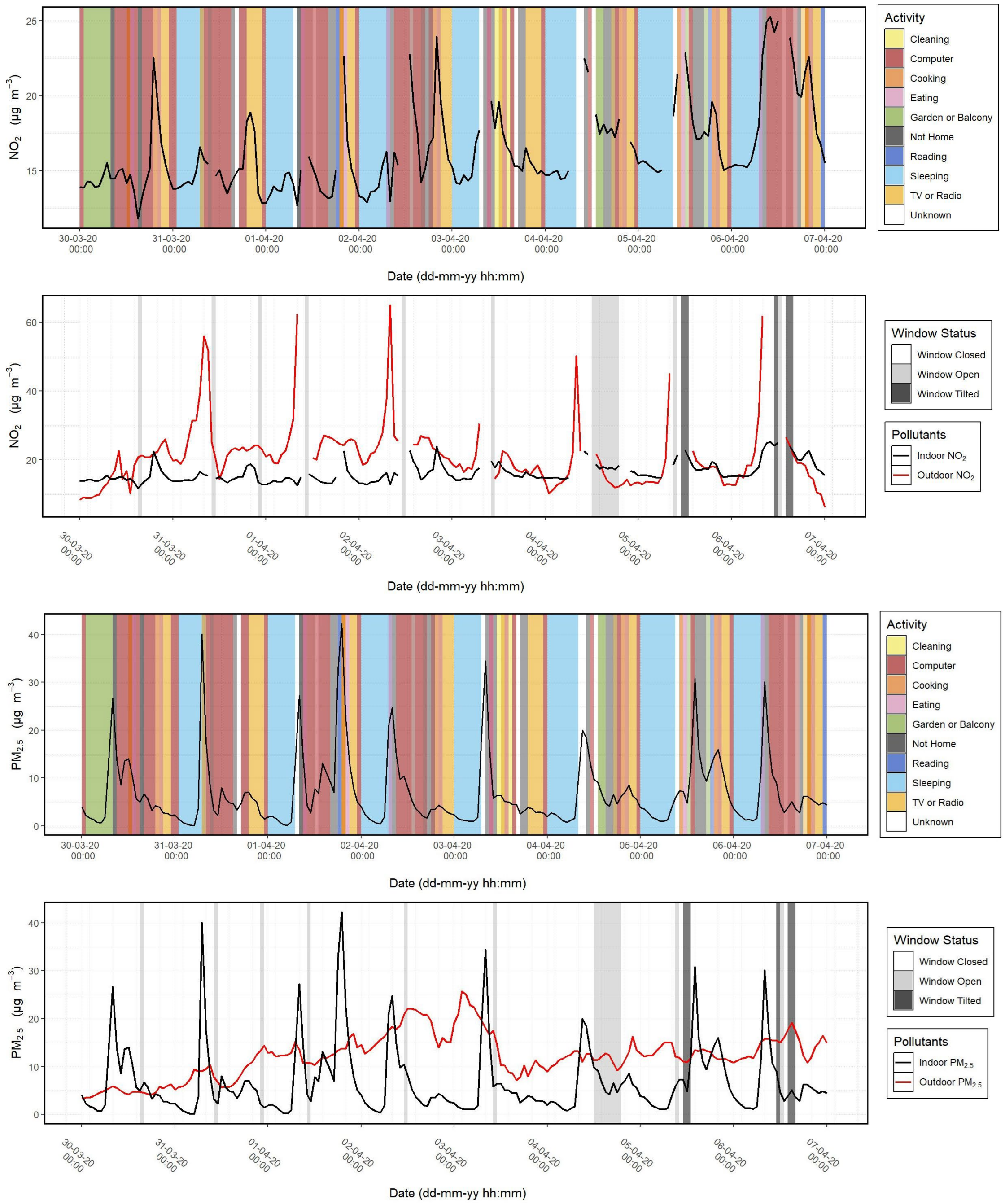

Figure S15. Time series of pollutant concentration combined with logged activities and window status, for patient 3, week 4.

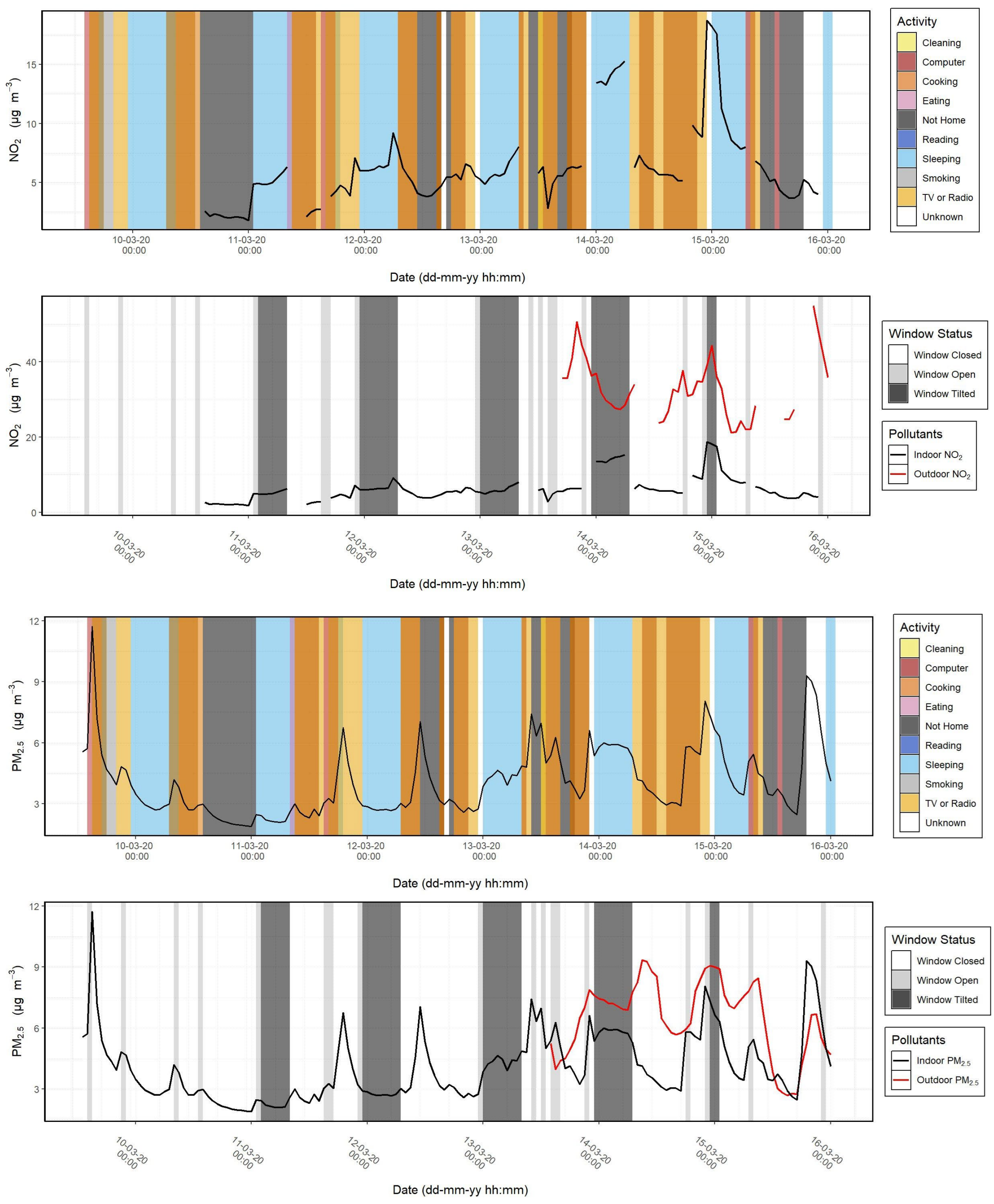

Figure S16. Time series of pollutant concentration combined with logged activities and window status, for patient 4, week 1.

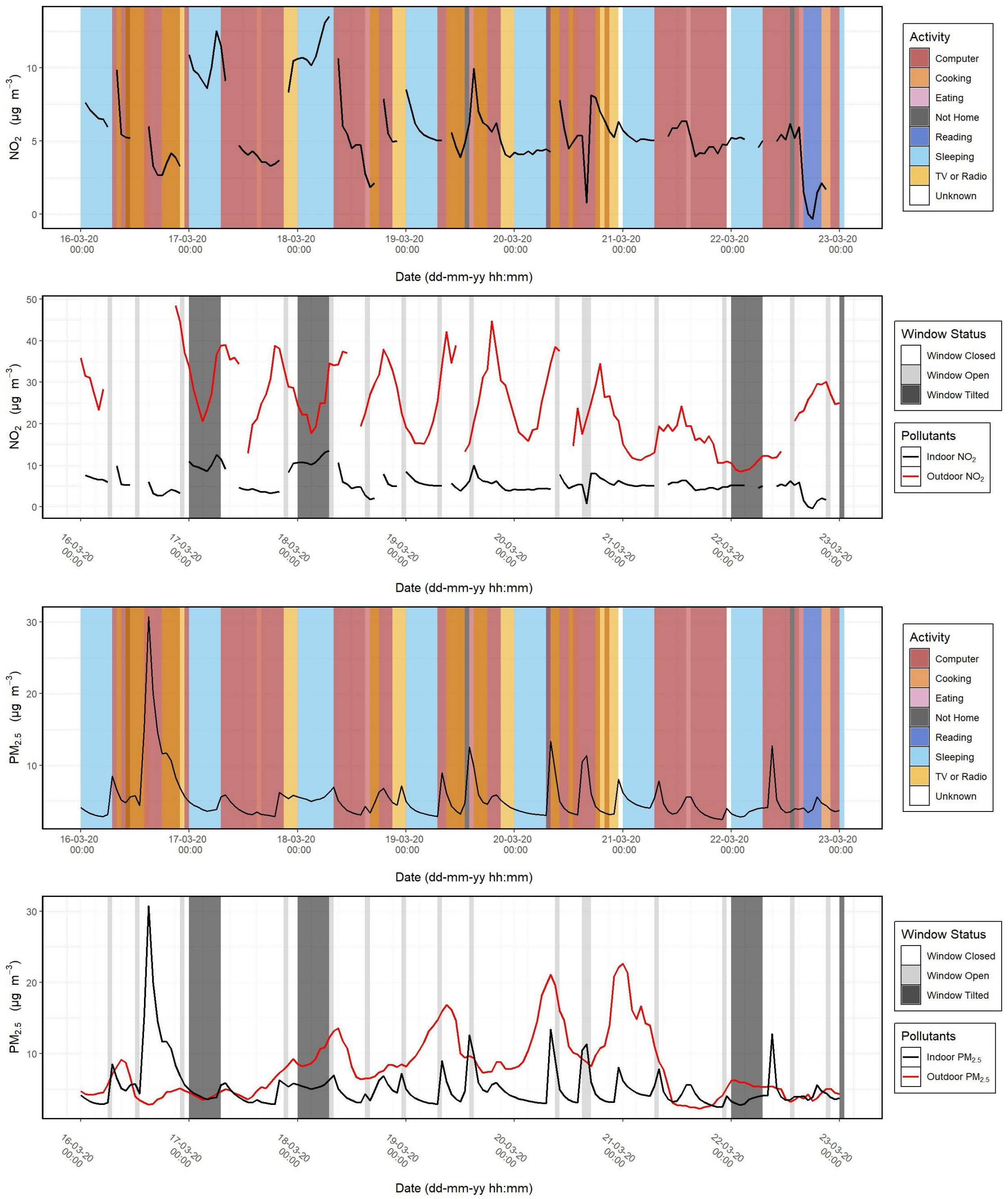

Figure S17. Time series of pollutant concentration combined with logged activities and window status, for patient 4, week 2.

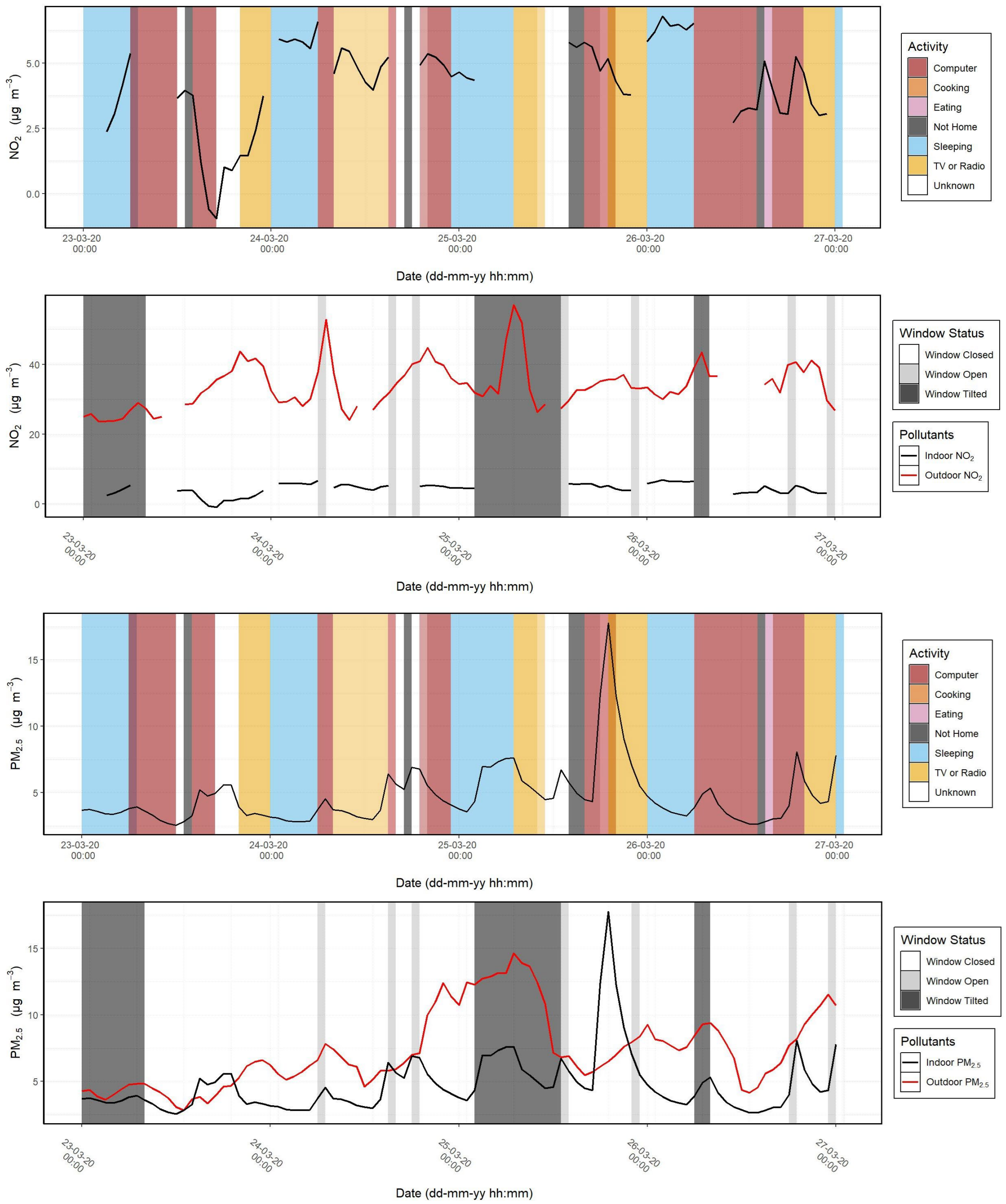

Figure S18. Time series of pollutant concentration combined with logged activities and window status, for patient 4, week 3.

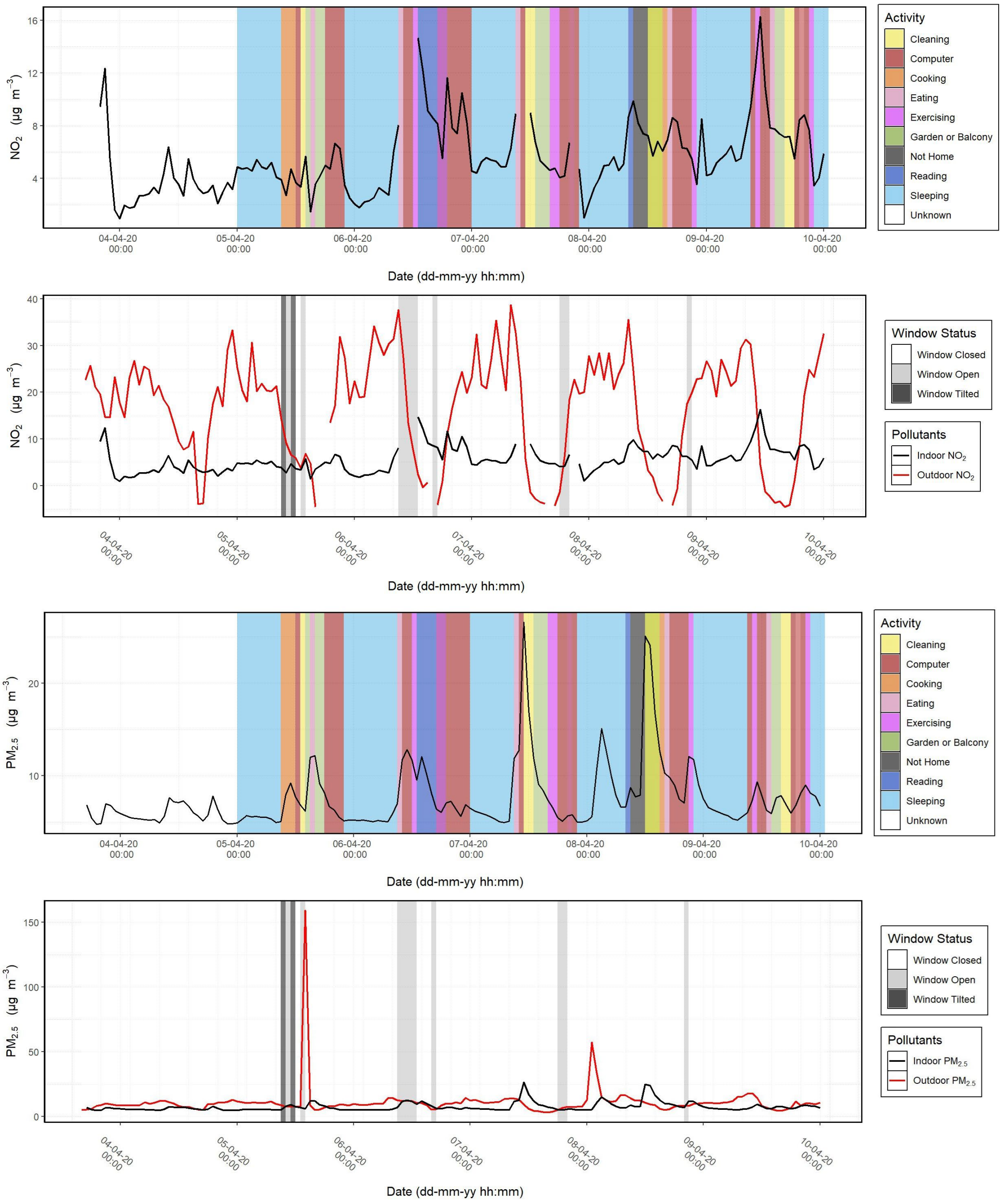

Figure S19. Time series of pollutant concentration combined with logged activities and window status, for patient 6, week 1.

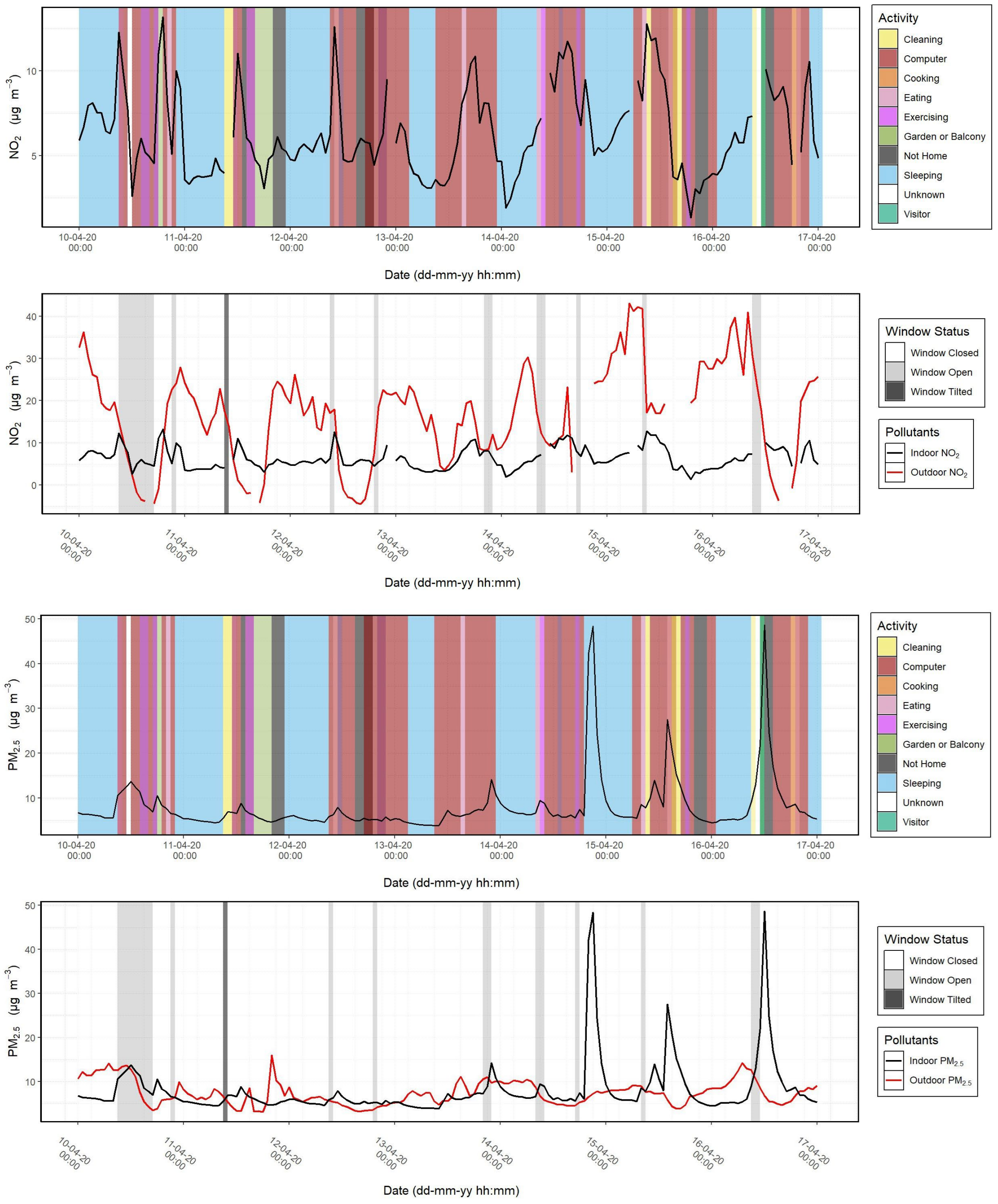

Figure S20. Time series of pollutant concentration combined with logged activities and window status, for patient 6, week 2.

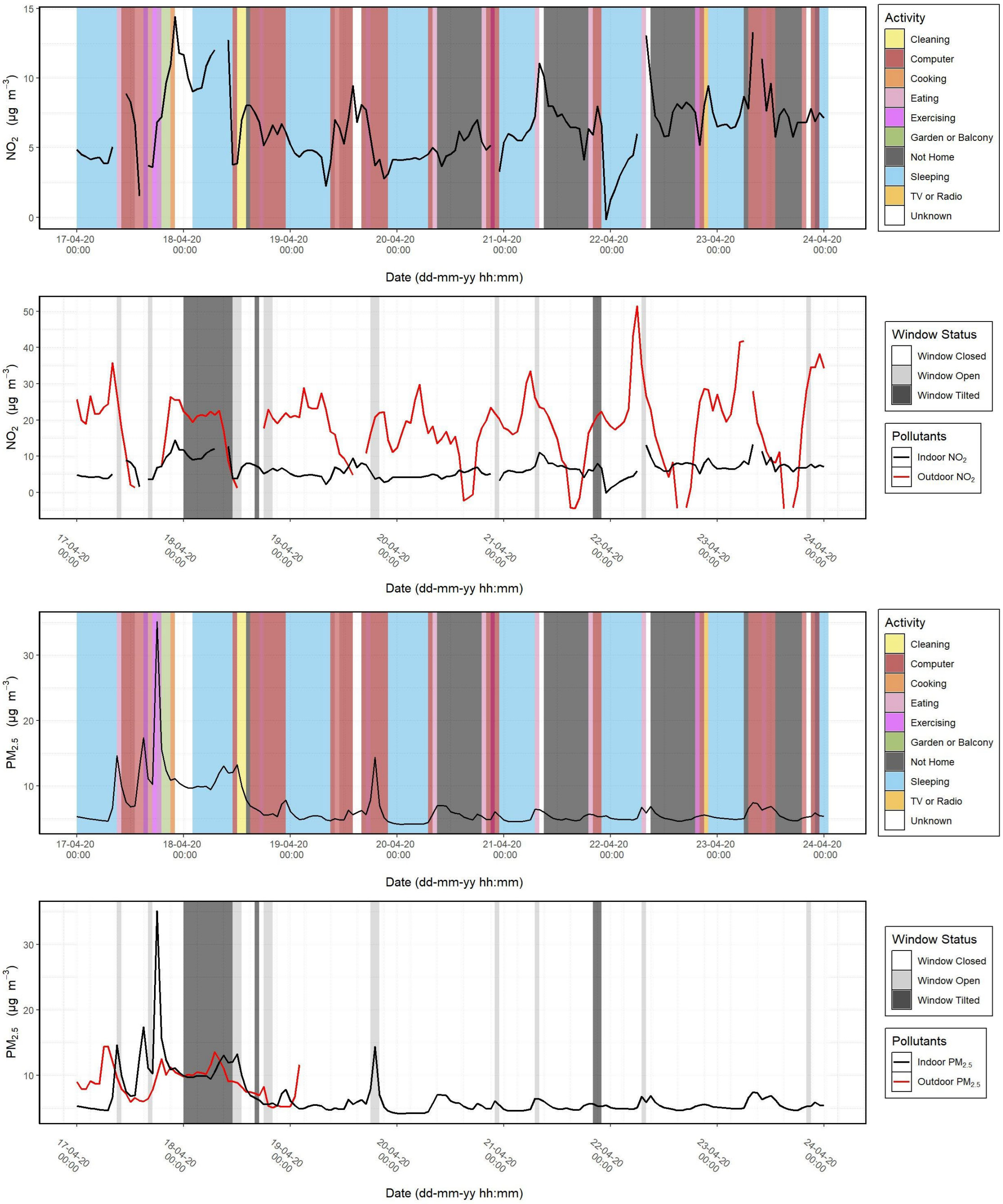

Figure S21. Time series of pollutant concentration combined with logged activities and window status, for patient 6, week 3. Note that the outdoor sensor stopped working on 19 April 2020 at 3:00 am.

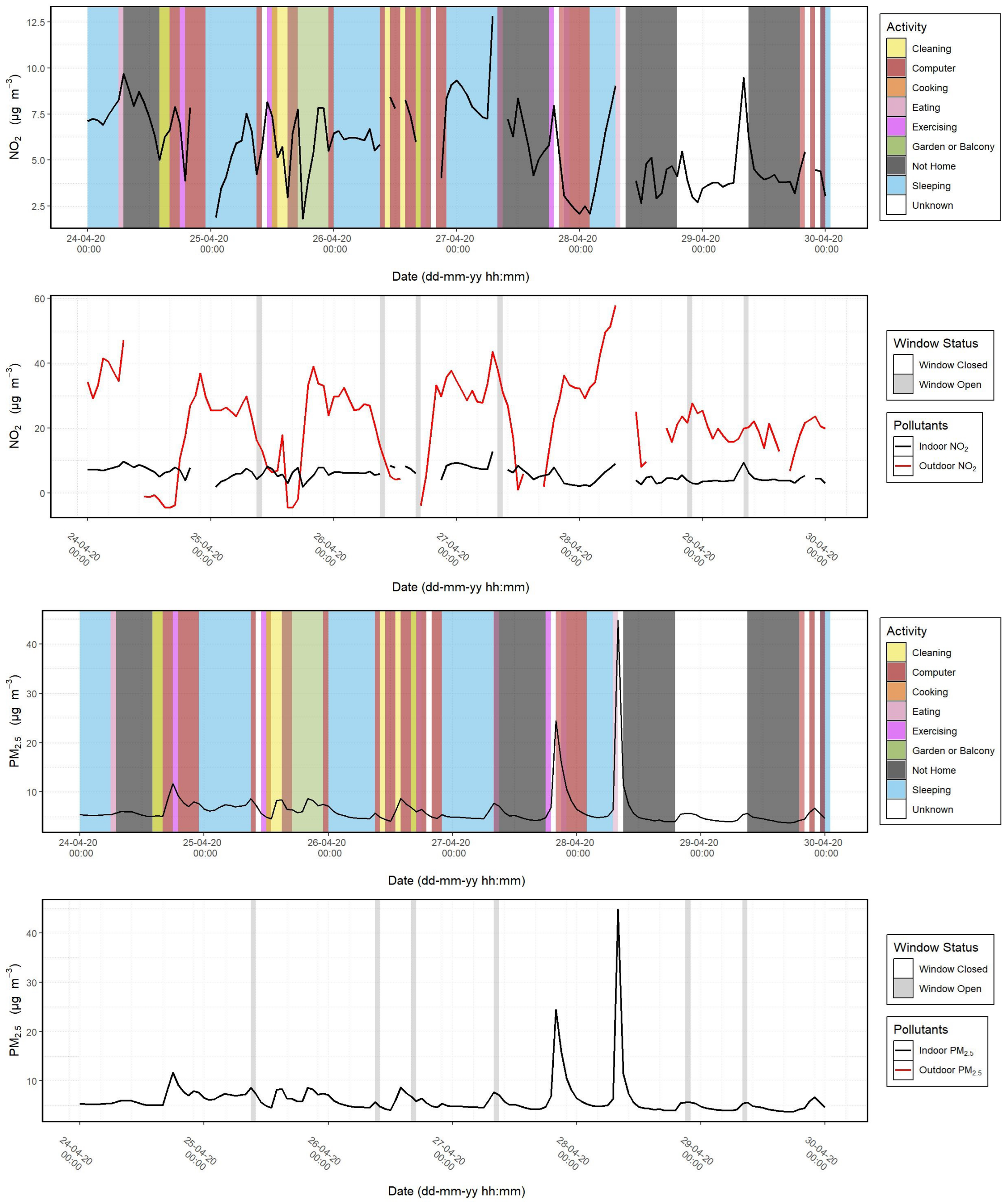

Figure S22. Time series of pollutant concentration combined with logged activities and window status, for patient 6, week 4.

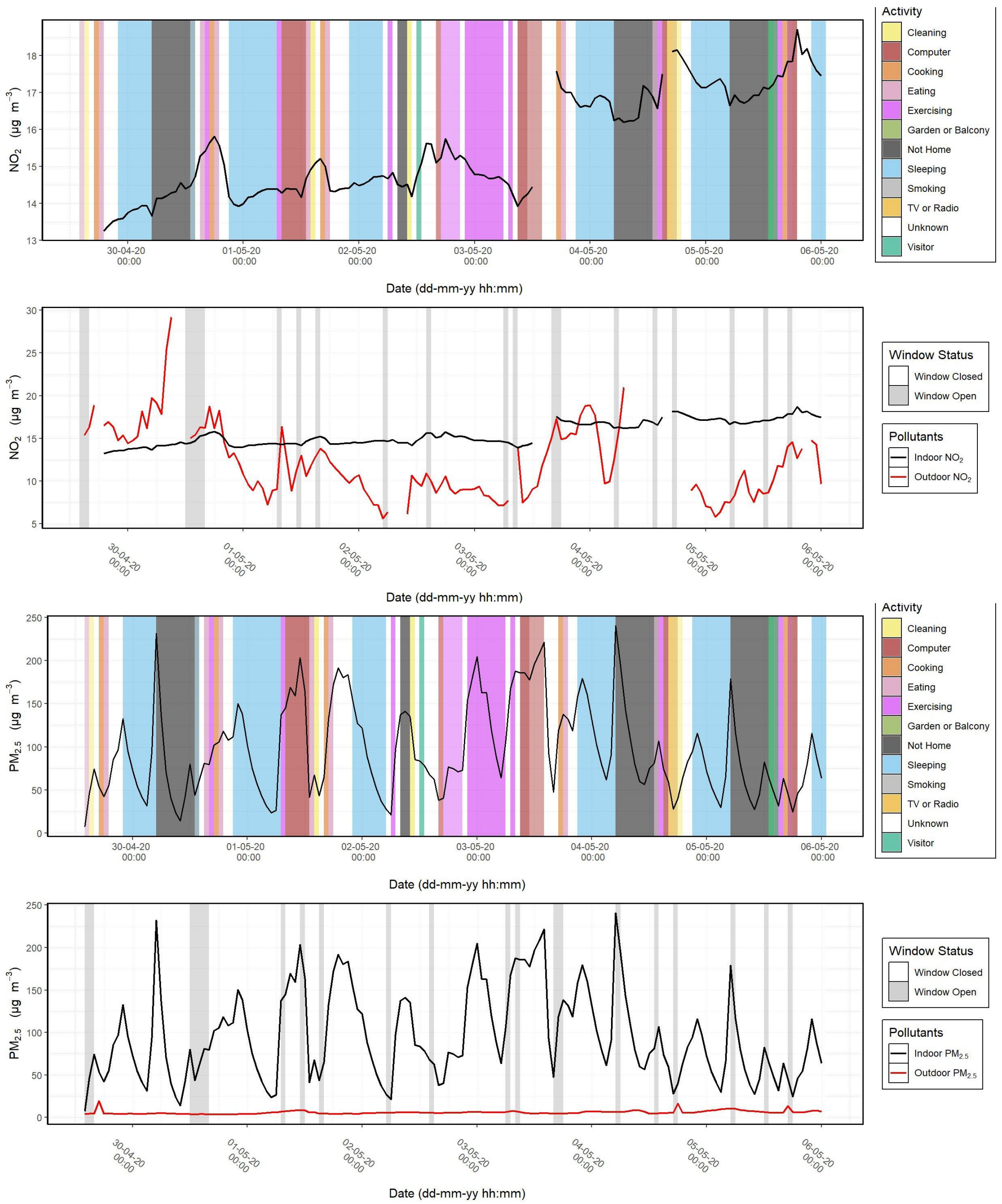

Figure S23. Time series of pollutant concentration combined with logged activities and window status, for patient 7, week 1.

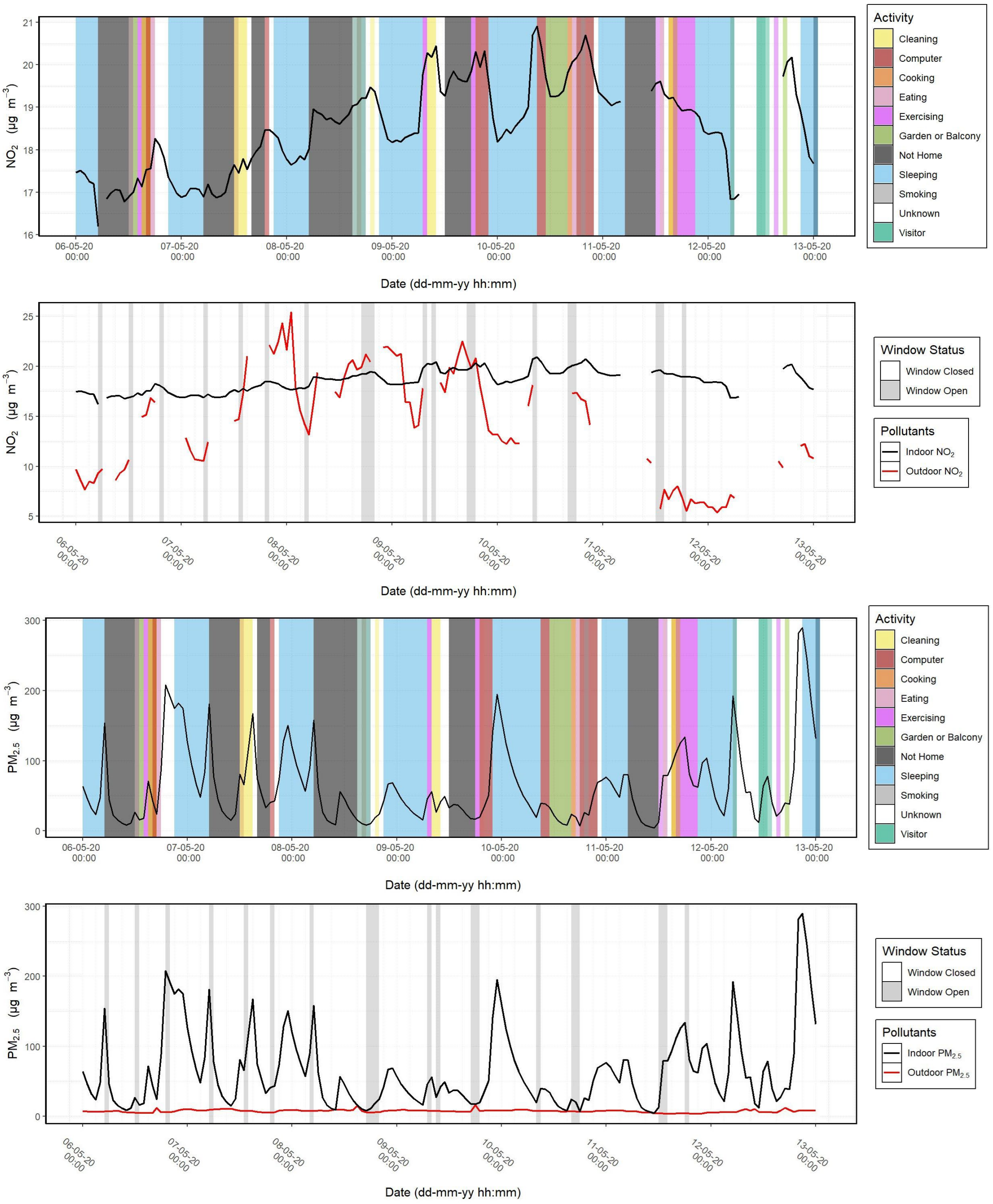

Figure S24. Time series of pollutant concentration combined with logged activities and window status, for patient 7, week 2.

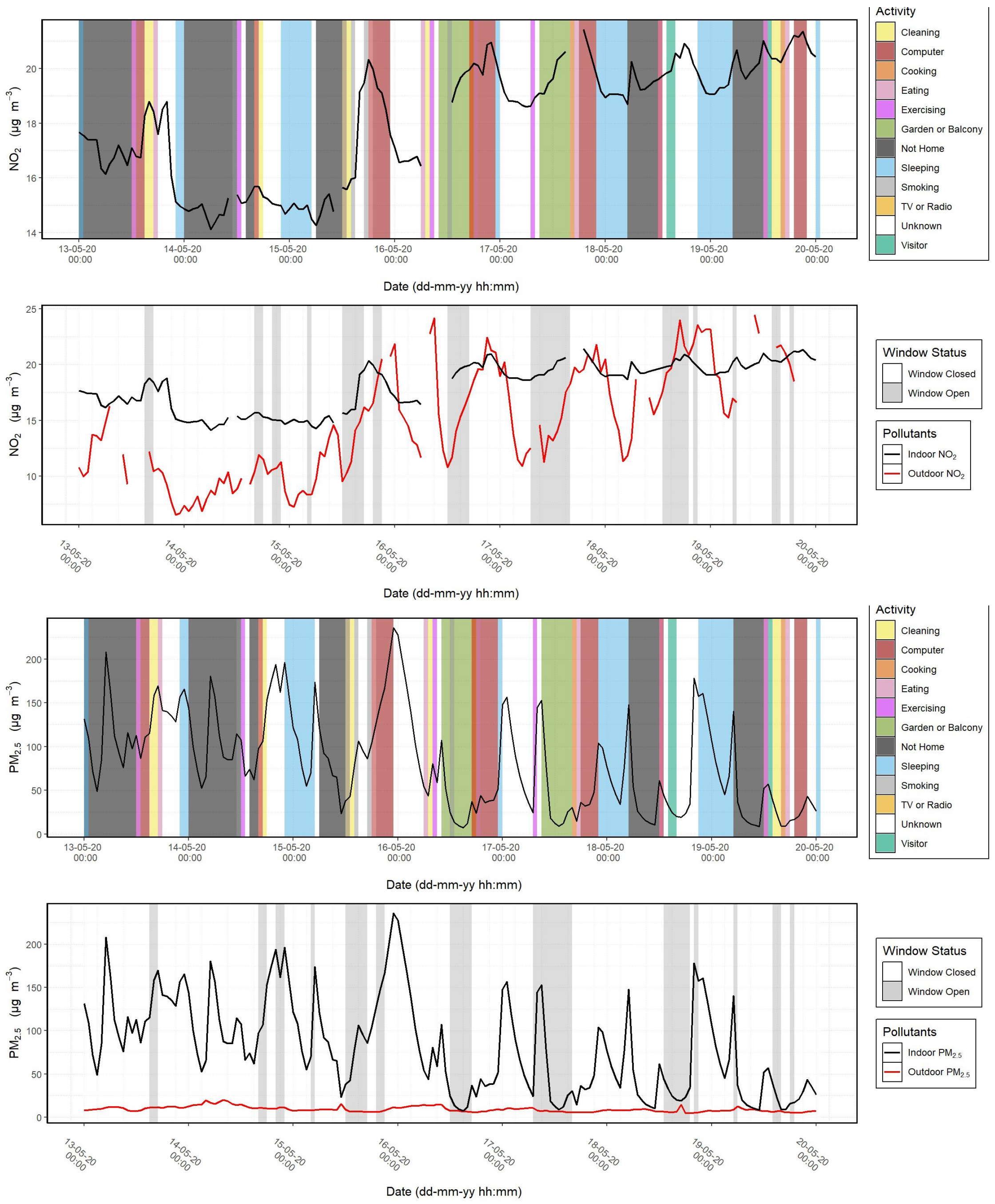

Figure S25. Time series of pollutant concentration combined with logged activities and window status, for patient 7, week 3.

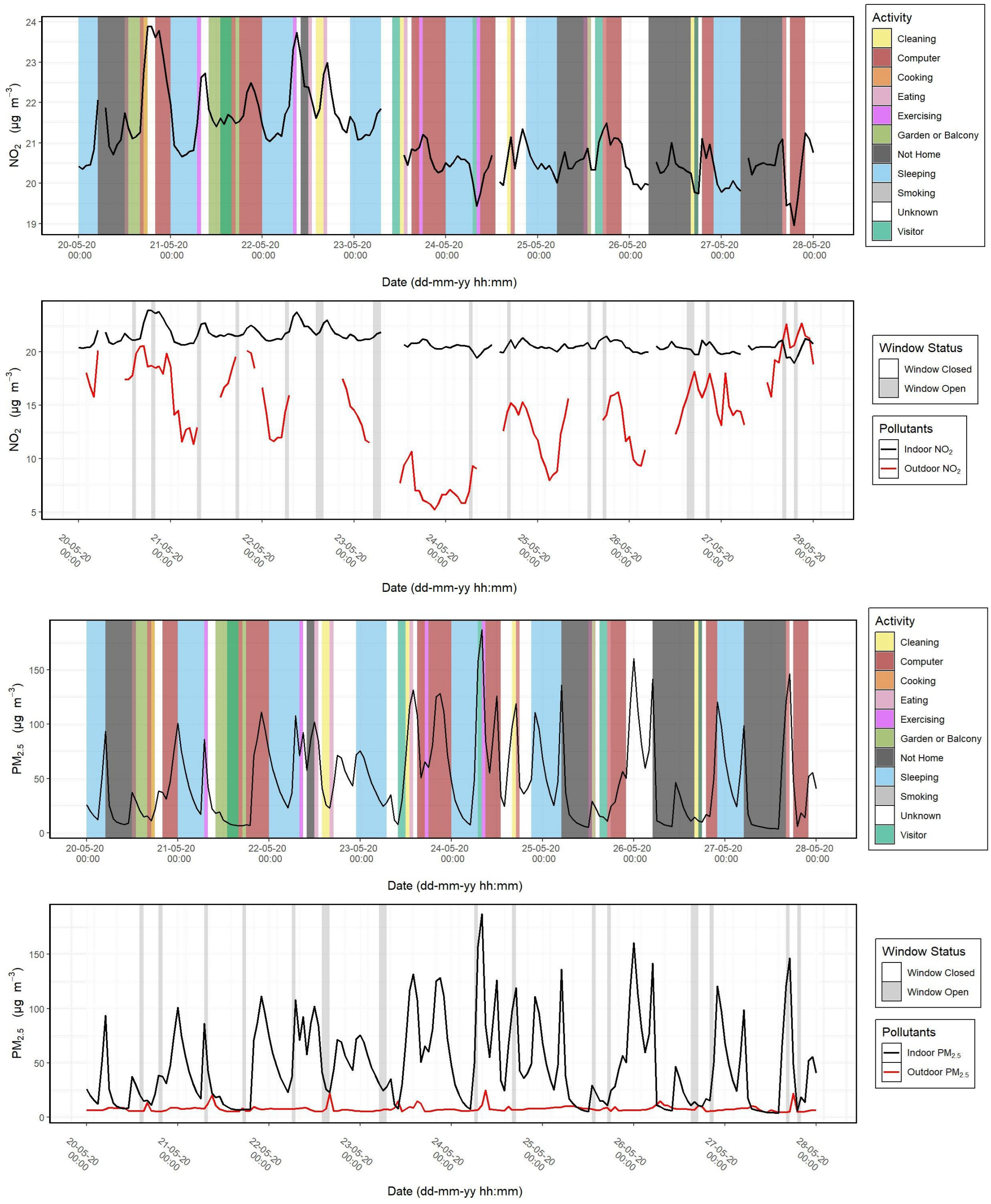

Figure S26. Time series of pollutant concentration combined with logged activities and window status, for patient 7, week 4.

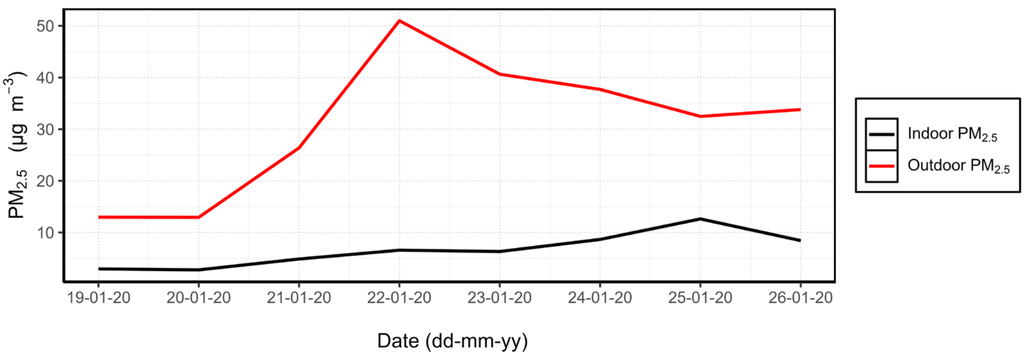

Figure S27. Daily indoor and outdoor $PM_{2.5}$ concentrations during PM alert (from 21st January to 26th January 2020) during the measurement campaign in the home of patient 1.

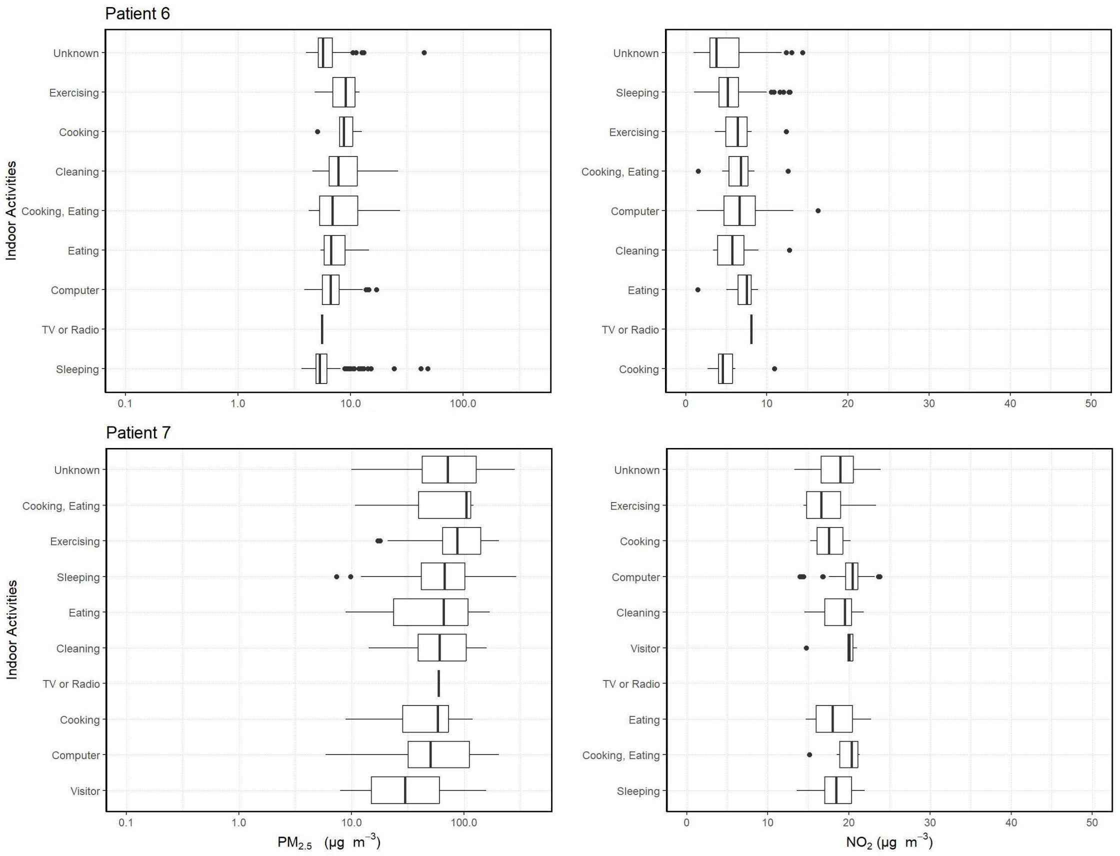

Figure S28. Individual activity specific $PM_{2.5}$ (left) and NO_2 (right) concentrations, for patients 6 and 7. Note that the $PM_{2.5}$ concentrations are on a logarithmic scale.

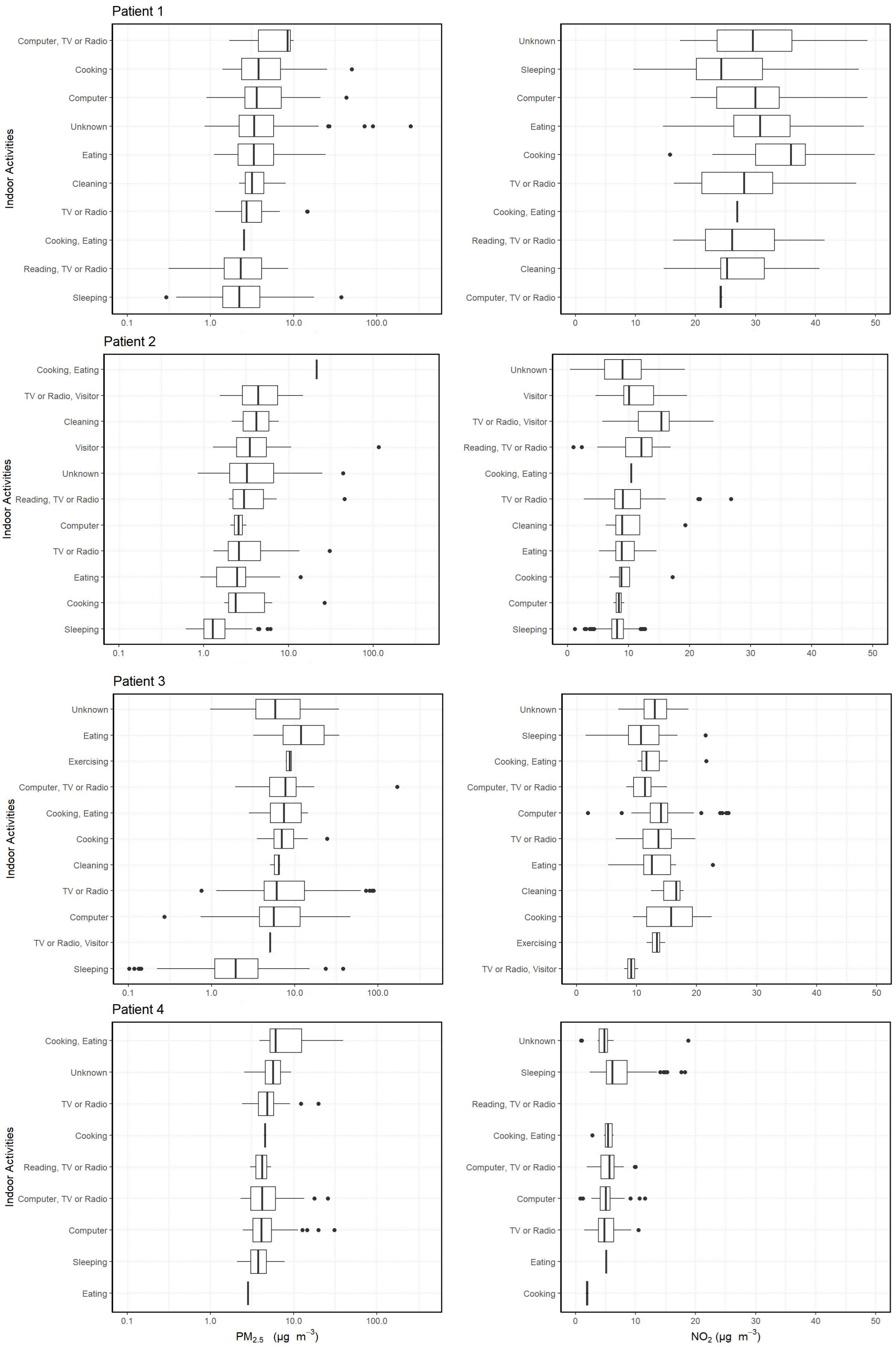

Figure S29. Individual activity specific $PM_{2.5}$ (left) and NO_2 (right) concentrations, for patients 1, 2, and 3 and 4. Note that the $PM_{2.5}$ concentrations are on a logarithmic scale.

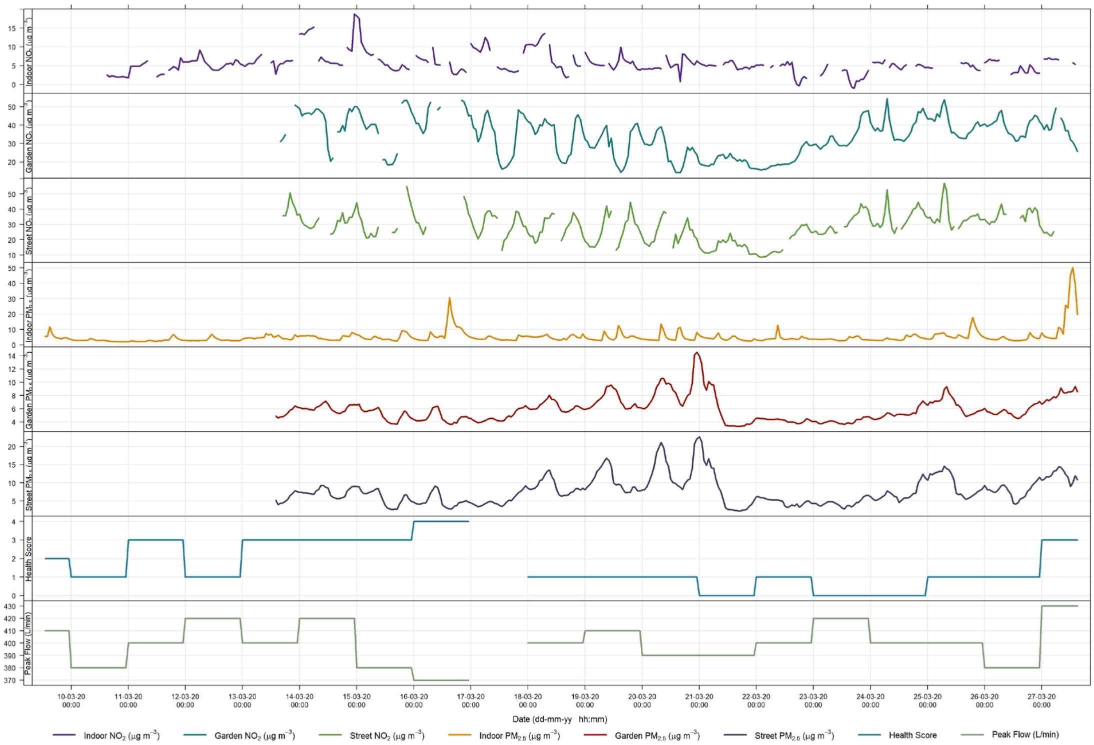

Figure S30. Hourly indoor and outdoor (garden and street) PM2.5 and NO2 concentrations, self-reported health score and PEF for patient 4.

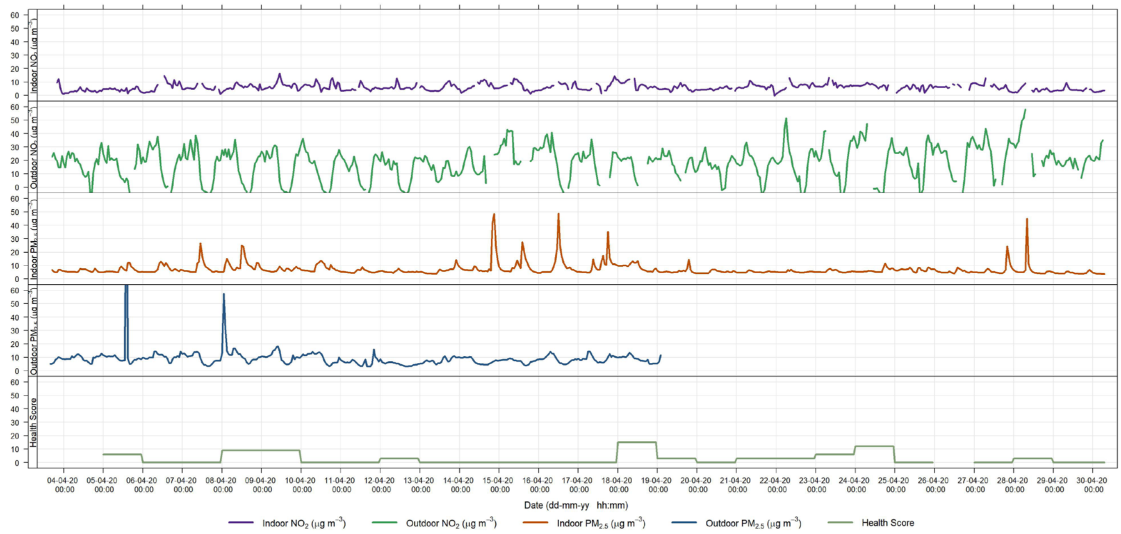

Figure S31. Hourly indoor and outdoor PM2.5 and NO2 concentrations, self-reported health score and PEF for patient 6. Note that the outdoor sensor stopped working on 19 April 2020 at 3:00 am.

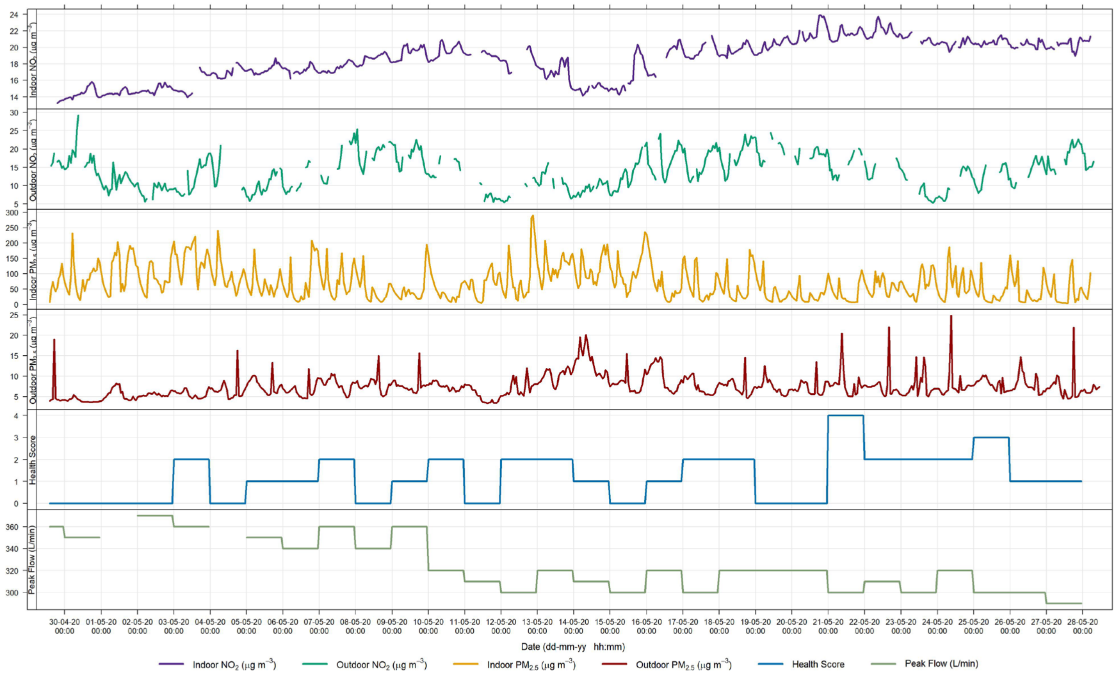

Figure S32. Hourly indoor and outdoor $PM_{2.5}$ and NO_2 concentrations, self-reported health score and PEF for patient 7.
